# Neutrophil Gelatinase–Associated Lipocalin Drives Cardiac Remodeling in Rats With Chronic Kidney Disease

**DOI:** 10.1161/HYPERTENSIONAHA.125.25658

**Published:** 2026-02-17

**Authors:** Matthieu Soulié, Tania Sánchez-Bayuela, Ixchel Lima-Posada, Yohan Stephan, Lionel Nicol, Zohra Lamiral, Jeremy Lagrange, Adriaan Voors, Natalia Lopez-Andres, Nicolas Girerd, Paul Mulder, Frédéric Jaisser

**Affiliations:** 1 Faculty of Medicine, Unité mixte de recherche Institute of Cardiometabolism and Nutrition (ICAN) 1166, Sorbonne Université, Paris, France (M.S., T.S.-B., I.L.-P., F.J.).; 2 Institut national de la santé et de la recherche médicale (INSERM), Endothelium, Valvulopathy & Heart Failure (ENVI) U1096, Normandie Université, Rouen, France (M.S., Y.S., L.N., P.M.).; 3 INSERM, Centre d’Investigations Cliniques-Plurithématique 1433, UMR 1116, Centre Hospitalier Régional Universitaire de Nancy, Investigation Network Initiative Cardiovascular and Renal Clinical Trialists, Université de Lorraine, France (Z.L., J.L., N.G., F.J.).; 4 Department of Cardiology, University Medical Centre Groningen, University of Groningen, the Netherlands (A.V.).; 5 Cardiovascular Translational Research, Navarrabiomed (Miguel Servet Foundation), Instituto de Investigación Sanitaria de Navarra, Hospital Universitario de Navarra, Universidad Pública de Navarra, Pamplona, Spain (N.L.-A.).

**Keywords:** chronic kidney failure, galectin 3, heart failure, lipocalin-2, mineralocorticoid receptor antagonists

## Abstract

**BACKGROUND:**

Patients with chronic kidney disease (CKD) are at high risk of cardiovascular complications. We have shown that Ngal (neutrophil gelatinase–associated lipocalin)/lcn2 is involved in aldosterone-induced cardiac remodeling and inflammation. Here, we investigated the role of Ngal in the progression of cardiorenal syndrome.

**METHODS:**

CKD was induced in rats via 5/6 nephrectomy in wild-type and Ngal knockout rats. Cardiorenal functions were assessed 3 months after subtotal nephrectomy or sham operation. Cardiac fibroblasts were isolated from wild-type rats and incubated with or without rNgal (recombinant Ngal) and Gal-3 (galectin-3).

**RESULTS:**

Cardiac perfusion was less impaired in CKD Ngal knockout than in CKD wild type. Left ventricle interstitial fibrosis was more severe in CKD wild type than in sham but was blunted in CKD Ngal knockout rats. Levels of Gal-3, Col1 (collagen 1), Ccl2 (C-C motif chemokine ligand 2), and IL-6 (interleukin-6) were high in cardiac fibroblasts incubated with rNgal. A similar pattern was observed in cells treated with recombinant Gal-3. Both Ngal and Gal-3 induced activation of the Tlr4 (toll-like receptor 4)-Myd88 (myeloid differentiation primary response 88) pathway. The effects of rNgal were blunted by concomitant treatment with Gal-3 or Tlr4 inhibitors, suggesting that Gal-3 contributes to Ngal-induced cardiac fibrosis and inflammation by activating the Tlr4-Myd88 pathway. In both MEDIA-DHF (Metabolic Road to Diastolic Heart Failure) and BIOSTAT-CHF (Biology Study to Tailored Treatment in Chronic Heart Failure) cohorts, elevated levels of Ngal and Gal-3 were associated with advanced diastolic dysfunction and adverse clinical outcomes, particularly among patients with impaired renal function.

**CONCLUSIONS:**

In CKD rats, Ngal was involved in cardiac remodeling via a Gal-3/Tlr4-dependent pathway, increasing inflammation and fibrosis, and correlated to cardiac outcomes in the MEDIA-DHF and BIOSTAT-CHF cohorts.

**Novelty and Relevance**

**What Is New?**

Rat *Ngal* (neutrophil gelatinase–associated lipocalin) gene inactivation reduces cardiac remodeling in the context of chronic kidney disease.

We describe a novel Ngal signaling pathway via Gal-3 (galectin-3)-Tlr4 (toll-like receptor 4) that could be targeted in chronic kidney disease–associated cardiovascular dysfunction.

High Ngal/Gal-3 levels in patients are predictive of a poorer cardiac outcome, particularly when associated with estimated glomerular filtration rate <60 mL/min per 1.73 m^2^.

**What Is Relevant?**

Ngal modulates the inflammation, fibrosis, and, ultimately, cardiac damage associated with chronic kidney disease.

**Clinical/Pathophysiological Implications?**

The development of novel therapeutic approaches to target organ damage, including extracellular matrix remodeling and inflammation, which are common underlying mechanisms of cardiac dysfunction associated with renal disease, will be a major goal over the next decade.

Chronic kidney disease (CKD) is characterized by a progressive deterioration of renal structure and function. Cardiac and renal functions are closely intertwined, as impaired kidney function exacerbates heart failure (HF) and vice versa. CKD leads to volume overload and hypertension, increasing cardiac workload, promoting hypertrophy, and ultimately resulting in HF with preserved ejection fraction (HFpEF), a frequent complication in patients with CKD.^1^

The renin-angiotensin-aldosterone system is activated in CKD, with angiotensin II and aldosterone promoting cardiac inflammation and fibrosis.^2–4^ High aldosterone levels are associated with a higher cardiovascular risk and HFpEF development^5–7^ and a higher risk of CKD progression.^8^ Patients with high aldosterone levels also display greater deleterious left ventricular (LV) remodeling.^9^ Treatment optimization is crucial to prevent CKD progression, reduce cardiovascular risks, and improve the quality of life of patients with CKD. Targeting the aldosterone pathway, in addition to renin-angiotensin system blockade, has proved effective for reducing both CKD progression and cardiovascular risk in patients with type 2 diabetes with renal failure.^10^ Newer nonsteroidal MR (mineralocorticoid receptor) antagonists, such as finerenone, prevent the progression of HFpEF in various patients, including those with impaired renal function.^11,12^ However, the exact mechanisms of these beneficial effects in CKD remain unclear. Moreover, the fear of hyperkalemia associated with aldosterone/MR blockade in patients with CKD, although better controlled with nonsteroidal MR antagonists such as finerenone, remains a barrier to the widespread use of MR antagonists. We previously identified Ngal (neutrophil gelatinase–associated lipocalin) as a downstream target of the aldosterone/MR pathway, demonstrating its essential role in the development of inflammation and fibrosis upon aldosterone/MR activation in the kidney and cardiovascular system.^13,14^ These findings identify a potential novel therapeutic target within the MR pathway not associated with a risk of hyperkalemia.

Beyond the role of the MR in cardiac remodeling during CKD, multiple mechanisms contribute to cardiovascular pathology. CKD is characterized by persistent inflammation and oxidative stress, which promote proinflammatory, profibrotic, and tissue remodeling processes in the heart. Tlr4 (toll-like receptor 4) activation has been directly associated with the development of cardiac fibrosis.^15^ We previously reported that inflammatory pathways such as Tlr4 signaling are upregulated in the kidney of experimental CKD models in response to MR activation.^16^ This has also been reported in circulating monocytes from patients with CKD.^17^ Indeed, kidney injury further promotes the release of damage-associated molecular patterns, such as HMGB1 (high-mobility group box 1).^18^ Of note, HMGB1 release has been linked to Ngal and Tlr4 signaling induction,^18^ as well as other proinflammatory pathways such as NLRP3 (NOD-like receptor family, pyrin domain containing 3) inflammasome activation, and impaired autophagy during HF.^19^

We investigated, in this study, the potential of Ngal inhibition for preventing the development of CKD-associated cardiovascular outcomes and the mechanisms involved, with specific attention to the Tlr4 pathway.

## Methods

### Data Availability

The data generated and analyzed during this study are available from the corresponding author upon reasonable request. A detailed methods section is provided in the Supplemental Material.

### Experimental Design

Experiments were approved by the Darwin Ethics Committee of Sorbonne Université (22207-2019010411586046). They were conducted according to Institut national de la santé et de la recherche médicale animal care guidelines in accordance with DIRECTIVE 2010/63/EU of the European Parliament. Animals were housed in a facility with a controlled climate and a 12-hour light/12-hour dark cycle, with free access to water and food.

CKD was induced by 5/6 subtotal nephrectomy in 10-week-old male Sprague-Dawley rats (Charles River): two-thirds of the right kidney was removed in a first intervention, with the left kidney removed 10 days later. Ngal knockout and wild-type (WT) littermates underwent the procedure simultaneously. In sham-operated rats, both kidneys were exposed, without resection. Ten days after the second intervention, plasma creatinine and urea determinations were performed to assess renal function. The generation of the Ngal knockout mutant line is described in the Supplemental Methods, and a schematic diagram of the in vivo experimental design is provided in Figure S1A.

### Study Populations: Metabolic Road to Diastolic HF Cohort and BIOSTAT-CHF Cohort

The MEDIA-DHF cohort (Metabolic Road to Diastolic HF; unique identifier: NCT02446327) was a multinational, multicenter (10 centers), observational study that enrolled 626 patients. Eligible patients had (1) acute decompensated HF, (2) a recent (within the last 60 days) hospital discharge for acute HF, or (3) chronic stable HF in an ambulatory setting. The primary end point was the composite of cardiovascular death or first hospitalization for HF within a 12-month period of follow-up.^20,21^ The BIOSTAT-CHF study (Biology Study to Tailored Treatment in Chronic HF; EudraCT 2010-020808-29) was a prospective, multicenter study conducted across 11 European countries, enrolling adult patients with new-onset or worsening HF, defined on the basis of symptoms and either an LV ejection fraction ≤40% or high levels of natriuretic peptides BNP (B-type natriuretic peptide) (>400 pg/mL or NT-proBNP [N-terminal pro-B-type natriuretic peptide]>2000 pg/mL). The primary outcome was the composite of all-cause mortality and rehospitalization for HF within a 2-year period of follow-up. Each component was also analyzed separately as a secondary outcome. For the analysis presented here, we considered only patients with HFpEF.^22^ Both studies adhered to the principles of the Declaration of Helsinki and were approved by the competent ethics committees. All participants gave written informed consent.

### Statistical Analysis

For preclinical and ex vivo studies, the results are presented as the mean±SEM. Differences in the means between groups were assessed by 2-way repeated-measures ANOVA comparing 4 groups, provided that the data were normally distributed for each group (Shapiro-Wilk normality test, in GraphPad Prism 8.0.1). Analyses were performed with GraphPad Prism 8.0.1, and differences were considered significant if *P*<0.05.

For clinical studies, statistical analyses were performed with SAS, version 9.4. We considered *P*<0.05 in 2-tailed tests to indicate statistical significance for main effects, whereas *P*<0.10 was considered significant for interaction terms. Results are presented as β coefficients for linear models and hazard ratios (HRs) for survival analyses, each with 95% CI. In the MEDIA-DHF cohort, linear regression models were used to evaluate the association between pulmonary arterial systolic pressure and biomarker levels (Ngal or Gal-3 [galectin-3]; BIOTECHNE, Minneapolis) while accounting for renal function status (estimated glomerular filtration rate [eGFR]<60 versus ≥60 mL/min per 1.73 m^2^) and potential interactions between biomarkers and renal function. Analyses were stratified by renal function and performed with 3 levels of adjustment (univariable adjustment and adjustment for age and sex) and fully adjusted models including adjustment for age, sex, body mass index, diabetes, and hypertension. The assumptions underlying linear regression were systematically verified. Cox proportional hazards models were used in both MEDIA-DHF and BIOSTAT-CHF to investigate the associations between biomarker levels (Ngal and Gal-3) and clinical outcomes, namely, cardiovascular death or cardiovascular hospitalization in the MEDIA cohort, and all-cause death and HF hospitalization in the BIOSTAT study. Interactions were assessed in models incorporating the biomarker concerned, renal function category, and their multiplicative interaction. The proportional hazards assumption was tested with covariates varying over time. For each outcome, we report crude, age- and sex-adjusted, and fully adjusted HRs.

## Results

### Cardiorenal Impacts of CKD in WT and Knockout Ngal Rats

We evaluated the renal and cardiovascular consequences of CKD induction by 5/6 nephrectomy. Renal dysfunction was similar in CKD Ngal knockout and CKD WT rats, with high plasma urea and creatinine levels, albuminuria, or low GFR. Renal fibrosis was also similar in the 2 strains upon CKD induction (Figure S2A through S2G).

Cardiac Ngal levels were higher in CKD WT rats than in the sham-operated WT group, whereas Ngal was undetectable in the sham-operated and CKD Ngal knockout groups, as expected, indicating efficient *lcn2* gene inactivation (Figure S1B). Cardiac echocardiography parameters in the CKD WT and knockout groups were similar to those in the corresponding sham-operated groups, with no significant difference between CKD WT and CKD Ngal knockout rats (Table S1). Fractional shortening was lower in CKD Ngal knockout rats than in Ngal sham-operated rats but not in CKD WT rats relative to sham-operated WT rats (Table S1). The cardiac output of CKD rats was similar to that of the corresponding sham-operated rats. (Table S1). LV and body weights were similar in all groups (Table S1).

**Figure 1.**
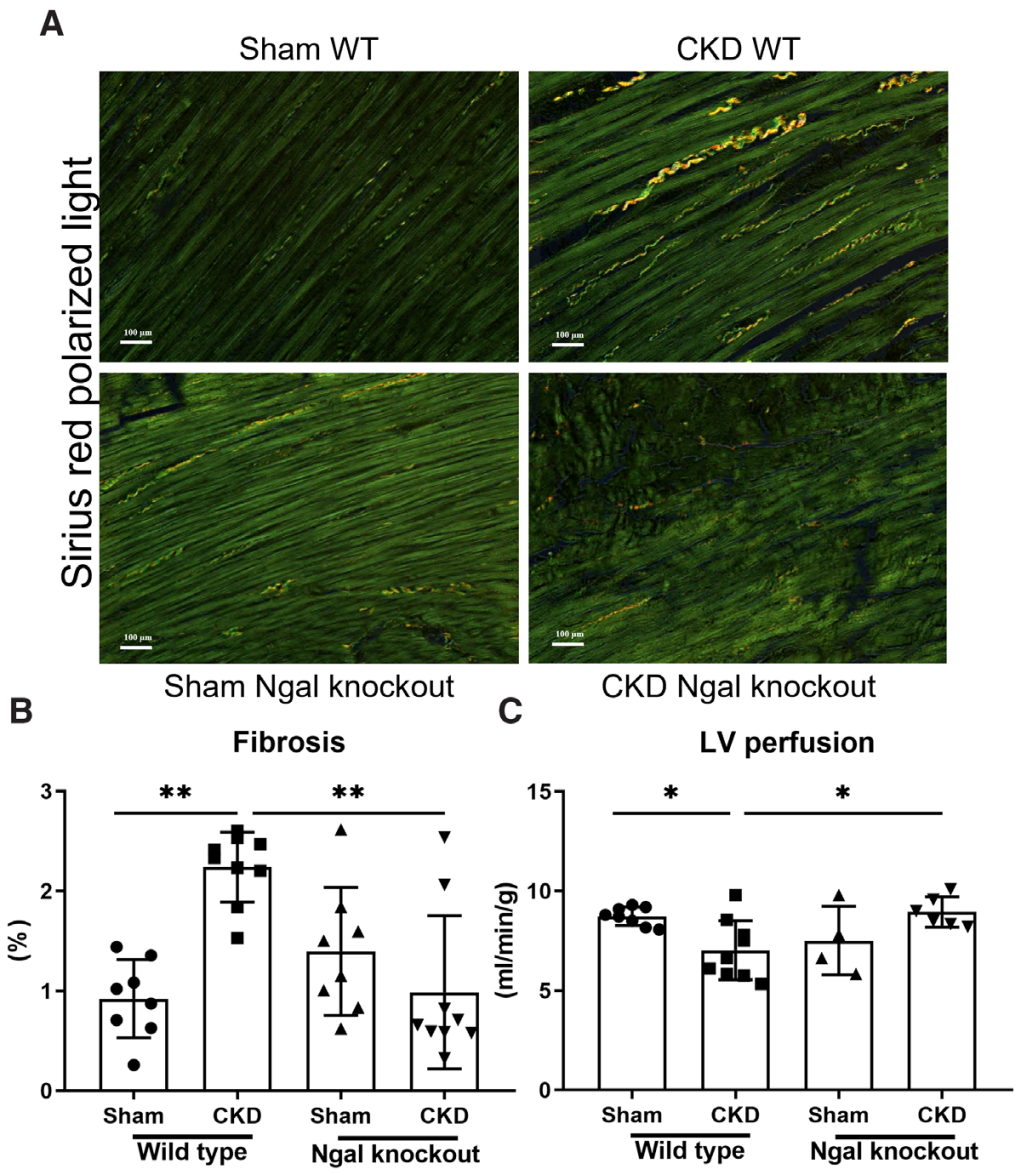
**Ngal (neutrophil gelatinase–associated lipocalin) knockout (KO) improves cardiac perfusion and fibrosis in rats with chronic kidney disease (CKD). A**, Cardiac fibrosis (illustration). **B**, Fibrosis (%). **C**, Myocardial perfusion (mL/min per g). Data are presented as the mean±SEM; n=8 to 10. Statistical analysis was performed by 1-way ANOVA followed by the Tukey post hoc test. LV indicates left ventricle; WT, wild type. *P<0.05, **P<0.01, and ***P<0.001.

LV interstitial fibrosis was more severe in CKD WT rats than in sham-operated WT rats (Figure 1A and 1B). However, the increase in cardiac fibrosis was less marked in CKD Ngal knockout rats than in CKD WT rats. (Figure 1A and 1B). LV perfusion was impaired in CKD WT rats relative to sham-operated WT rats (Figure 1C). LV cardiac perfusion in CKD Ngal knockout rats was greater than that in CKD WT rats (Figure 1C).

### Aldosterone Induces the Expression of Profibrotic and Inflammatory Genes, Including *Ngal* and *Gal-3*, in Rat Cardiac Fibroblasts, an Effect Abolished by Cotreatment With Finerenone

We previously reported that aldosterone increases the levels of profibrotic and proinflammatory markers in human cardiac fibroblasts (CFs), an effect blunted by the nonsteroidal MR antagonist finerenone.^13^ We obtained similar results in rat CFs, making it possible to perform further mechanistic studies in rat CFs. Relative levels of fibrosis and inflammation marker gene expression (*Col1a1*, *Col3a1*, *Ccl2* [C-C motif chemokine ligand 2], and *IL-6* [interleukin-6]) were increased by aldosterone in rat CFs, an effect abolished by finerenone (Figure S5A through S5D). Expression of the *Ngal* and *Gal-3* genes was also increased by aldosterone treatment in rat CFs, an effect blunted by finerenone (Figure S5E and S5F).

### Recombinant Ngal and Gal-3 Induce Fibrotic and Inflammatory Markers in CFs

**Figure 2.**
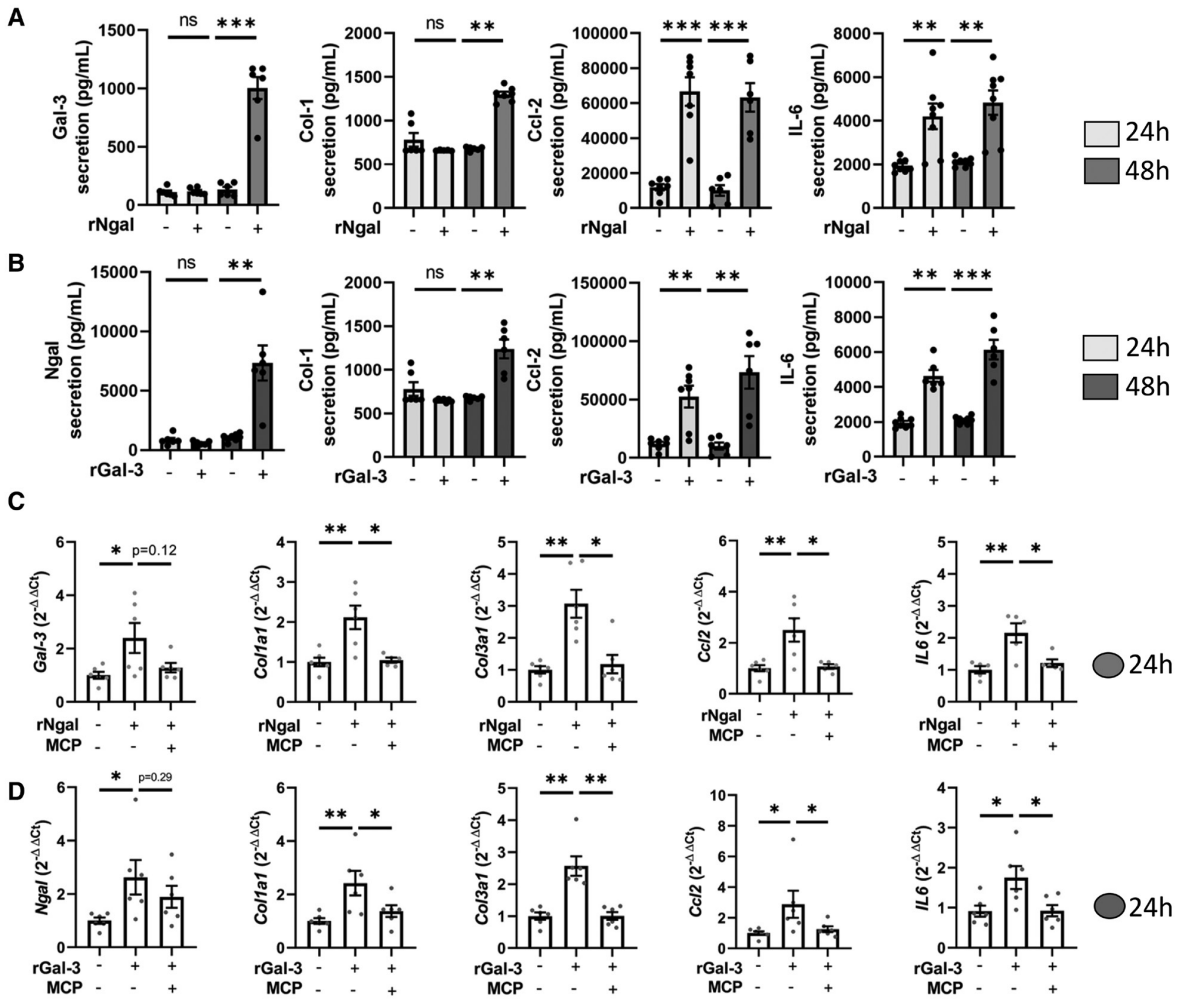
**Recombinant Ngal (neutrophil gelatinase–associated lipocalin) and Gal-3 (galectin-3) induce fibrotic and inflammatory proteins in a Gal-3 pathway–dependent manner in cardiac fibroblasts (CFs). A**, Col1 (collagen 1; pg/mL), Ccl2 (C-C motif chemokine ligand 2; pg/mL), IL-6 (interleukin-6; pg/mL), and Gal-3 secretion by CFs incubated with rNgal (recombinant Ngal; 500 ng/mL) for 24/48 hours. **B**, Col1 (pg/mL), Ccl2 (pg/mL), IL-6 (pg/mL), and Ngal (pg/mL) secretion by CFs incubated with rGal-3 (recombinant Gal-3; 5 µg/mL) for 24/48 hours. **C**, Expression (ΔΔCt values) of *Gal-3*, *Col1a1*, *Col3a1*, *Ccl2*, and *IL-6* genes in CFs incubated for 24 hours with rNgal (500 ng/mL)±modified citrus pectin (MCP; 500 µg/mL), a Gal-3 inhibitor. **D**, Expression (ΔΔCt values) of *Ngal*, *Col1a1*, *Col3a1*, *Ccl2*, and *IL-6* genes in CFs incubated with rGal-3 (5 µg/mL)±MCP (500 µg/mL) for 24 hours. Data are presented as the mean±SEM; n=5 to 6 biological replicates. Statistical analysis was performed by 1-way ANOVA followed by the Tukey post hoc test. *P<0.05, **P<0.01, and ***P<0.001.

rNgal (recombinant Ngal) increased the secretion of Ccl2 and IL-6 into the supernatant of CFs after 24 hours of exposure and the secretion of Col1 (collagen 1) and Gal-3 after 48 hours of exposure (Figure 2A). Parallel effects were observed with rGal-3 (recombinant Gal-3), which increased Ccl2 and IL-6 secretion at 24 hours, as well as Ngal and Col1 levels at 48 hours (Figure 2B). Both rNgal and rGal-3 also upregulated the expression of key genes involved in fibrosis and inflammation including *Gal-3*, *Ngal*, *Col1a1*, *Col3a1*, *Ccl2*, and *IL-6* with the most robust responses observed at concentrations of 500 ng/mL for rNgal and 10^−8^ M (5 μg/mL) for rGal-3, and at the 24-hour time point, those were the settings chosen for the following experiments (Figure 2C and 2D; Figure S4). As expected, coincubation with the Gal-3 inhibitor modified citrus pectin (MCP) blunted the increase in gene expression observed after coincubation with rGal-3 (Figure 2D). Importantly, coincubation with MCP, a Gal-3 inhibitor, also blunted the effects of rNgal, highlighting the crucial role of Gal-3 in the profibrotic and proinflammatory effects of Ngal (Figure 2C).

### The Profibrotic and Proinflammatory Effects of Ngal and Gal-3 in CFs Are Mediated by the Tlr4-Myeloid Differentiation Primary Response 88-Nuclear Factor-Kappa B Innate Immunity Pathway

**Figure 3.**
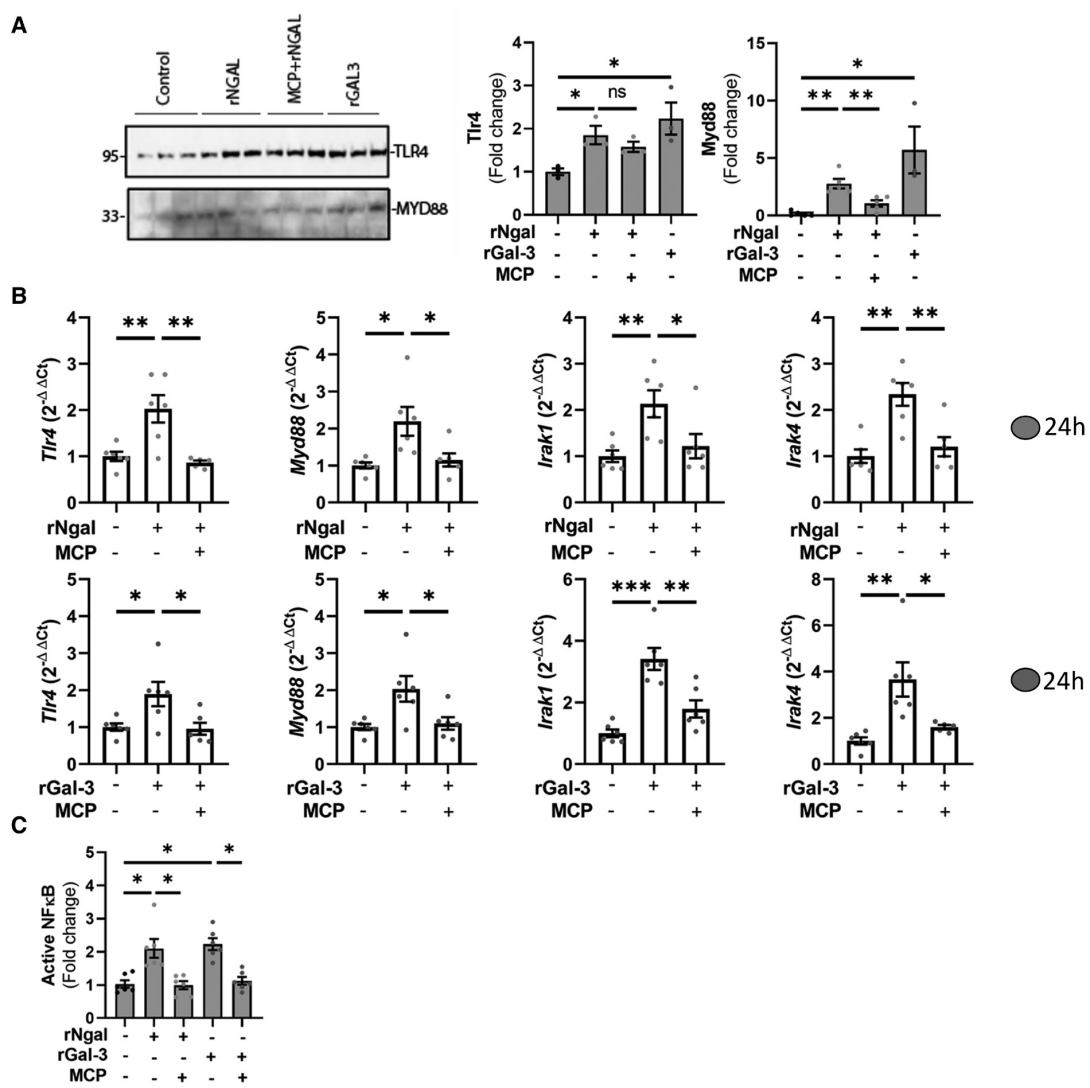
**Gal-3 (galectin-3)–dependent activation of Tlr4 (toll-like receptor 4)/Myd88 (myeloid differentiation primary response 88)/NF-κB (nuclear factor-kappa B) signaling by recombinant Ngal (neutrophil gelatinase–associated lipocalin) and Gal-3. A**, Protein levels of Tlr4 and Myd88 in cardiac fibroblasts (CFs) treated with rNgal (recombinant Ngal; 500 ng/mL)/rGal-3 (recombinant Gal-3; 5 µg/mL)±modified citrus pectin (MCP; 500 µg/mL) for 24 hours. **B**, Gene expression of Tlr4, Myd88, Irak (interleukin 1 receptor-associated kinase) 1, and Irak4 in CFs treated with rNgal (500 ng/mL)/rGal-3 (5 µg/mL)±MCP (500 µg/mL). **C**, NF-κB activation measured by a consensus sequence–based ELISA after 24 hours of treatment with the indicated stimuli. Protein levels are presented as fold change relative to untreated controls. Transcript levels were normalized to *Gapdh* and expressed relative to the control. Data are shown as mean±SEM; n=5 to 6 biological replicates. Statistical analysis was performed using 1-way ANOVA followed by the Tukey post hoc test. *P<0.05, **P<0.01, and ***P<0.001.

Both rNgal and rGal-3 increased the expression, at both mRNA and protein levels, of the innate immune response genes *Tlr4* and *Myd88* (myeloid differentiation primary response 88; Figure 3A and 3B), as well as the transcriptional activity of the nuclear transcription factor NF-κB (nuclear factor-kappa B; Figure 3C). Although Gal-3 and Tlr4 LV protein levels were not reduced in CKD Ngal knockout relative to CKD WT, the downstream Tlr4 effector Myd88 showed decreased expression in CKD Ngal knockout (Figure S3A through S3C). Both rNgal and rGal-3 also upregulated downstream components of Tlr4 signaling, including Irak (interleukin 1 receptor-associated kinase) 1 and Irak4 (Figure 3B). The expression of other *Tlrs* signaling through MyD88 was assessed and found to be low, with the exception of *Tlr5* (Figure S6). However, neither rNgal nor rGal-3 altered Tlr5 expression (Figure S6).

**Figure 4.**
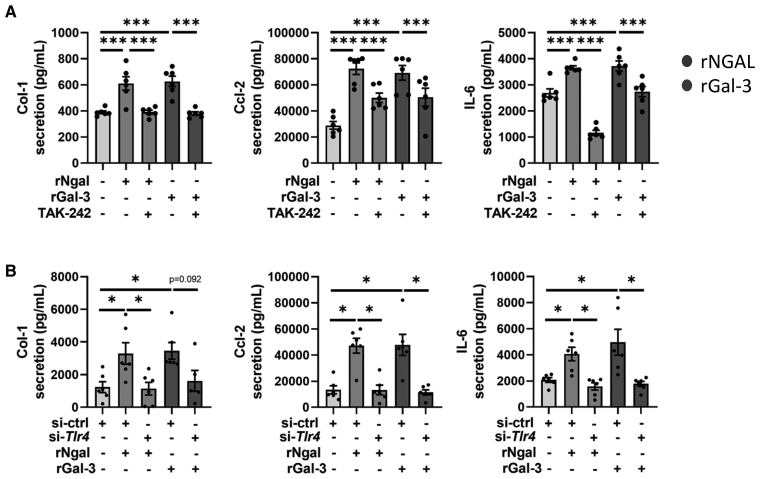
**Tlr4 (toll-like receptor 4) signaling is required for rNgal (recombinant neutrophil gelatinase–associated lipocalin) and rGal-3 (recombinant galectin-3)–driven inflammatory and fibrotic responses. A**, Col1 (collagen 1; pg/mL), Ccl2 (C-C motif chemokine ligand 2; pg/mL), and IL-6 (interleukin-6; pg/mL) secretion in cardiac fibroblasts (CFs) incubated with rNgal (500 ng/mL)/rGal-3 (5 µg/mL) without Tlr4 inhibitor TAK-242 (Tlr4 inhibitor; 1 µmol/L) for 48 hours. **B**, Col1 (pg/mL), Ccl2 (pg/mL), and IL-6 (pg/mL) secretion in CFs incubated with rNgal (500 ng/mL)/rGal-3 (5 µg/mL) without *Tlr4* gene silencing with small interfering (si)-RNA for 48 hours. Target transcript expression was normalized to *Gapdh* and presented relative to the control. Data are shown as mean±SEM; n=5 to 6 biological replicates. Statistical analysis was performed using 1-way ANOVA followed by the Tukey post hoc test. *P<0.05, **P<0.01, and ***P<0.001.

The activation of the Tlr4 pathway induced by rNgal and rGal-3 was inhibited by the Gal-3 inhibitor MCP (Figure 3A through 3C). As expected, the induction of Tlr4, Myd88, Irak1, and Irak4 was blunted by cotreatment with TAK-242 (Tlr4 inhibitor), which achieved ≈65% knockdown efficiency of Tlr4 expression, in CFs exposed to rNgal or rGal-3 (Figures S7A, S8A, and S8B). Inhibition of the Tlr4 pathway with either TAK-242 or si-Tlr4 also reduced the increases in Col1, Ccl2, and IL-6 secretion (Figure 4A and 4B), as well as the upregulation of *Col1a1*, *Col3a1*, *Ccl2*, and *IL-6* gene expression (Figures S7B and S8C) triggered by rNgal and rGal-3. Together, these findings demonstrate that the Tlr4-Myd88 pathway plays a central role in Ngal-Gal-3-mediated signal transduction, driving profibrotic and proinflammatory responses in CFs.

### Correlation Between Ngal, Gal-3, and Cardiovascular Outcomes in the MEDIA-DHF and BIOSTAT-CHF Cohorts

In patients with HFpEF from the MEDIA-DHF and BIOSTAT-CHF cohorts, Ngal and Gal-3 levels were moderately correlated. In MEDIA-DHF, the Spearman correlation coefficient for the relationship between Ngal and Gal-3 (Olink) was 0.27 (0.19–0.36; *P*<0.0001). In BIOSTAT-CHF, Ngal was more strongly correlated with Gal-3 determinations by mass spectrometry (r=0.53) than with those obtained with Olink (r=0.16; both *P*<0.0001), suggesting platform-dependent variation.

On stratification for renal function (eGFR <60 versus ≥60 mL/min per 1.73 m^2^), both biomarkers were found to display differential association with pulmonary arterial systolic pressure, a marker of advanced diastolic dysfunction (Table 1). In MEDIA-DHF, Ngal was significantly associated with pulmonary arterial systolic pressure only in patients with low eGFR values (β=3.08 [[95% CI, 0.93–5.24] mm Hg; *P*=0.005), with no association found in patients with preserved renal function (β=−0.16 mm Hg; *P*=0.95). Similarly, high Gal-3 levels were associated with a higher pulmonary arterial systolic pressure only in patients with an eGFR <60 (β=6.76 [[95% CI, 1.95–11.57] mm Hg; *P*=0.006), not in those with an eGFR ≥60 (β=3.28 mm Hg; *P*=0.15).

**Table 1.**
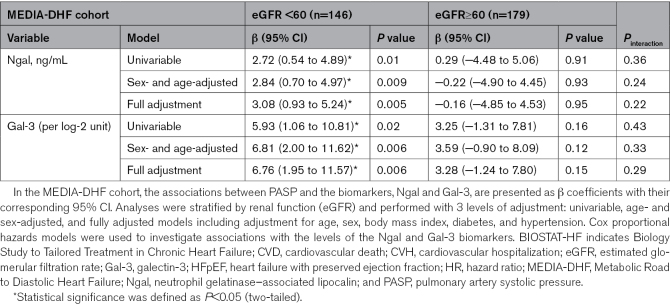
Association of Ngal/Gal-3 Levels With PASP in Patients With HFpEF From the MEDIA-DHF Cohort According to Renal Function (<60 or ≥60 mL/min per 1.73 m^2^)

A similar pattern was observed for clinical outcomes (Table 1). In MEDIA-DHF, high Gal-3 levels were associated with a higher risk of cardiovascular hospitalization or death in the eGFR <60 group (HR, 2.18 [95% CI, 0.95–5.00]; *P*=0.07; age- and sex-adjusted; HR, 2.21 [95% CI, 0.95–5.15]; *P*=0.07; fully adjusted) but not in the eGFR ≥60 group (HR, 0.72 and 0.73; both *P*>0.5), with an interaction of borderline significance (*P*=0.09). Ngal was not significantly associated with outcomes regardless of renal function.

In BIOSTAT-CHF, Ngal was associated with all-cause death in patients with an eGFR <60 (HR, 1.60 [95% CI, 1.16–2.19]; *P*=0.004) but not in those with an eGFR ≥60 (HR, 0.63; *P*=0.37), with an interaction of borderline significance (*P*=0.09 and 0.08 in age- and sex-adjusted and fully adjusted models, respectively). Gal-3 levels determined by mass spectrometry followed a similar pattern: significant association with all-cause death in the eGFR <60 group (HR, 1.27 per 10 ng/mL; *P*=0.004) with no significant association in the group with preserved eGFR (*P*=0.72), with an interaction *P*=0.04 (age- and sex-adjusted) and 0.08 (fully adjusted). Similar results were obtained for Gal-3 levels measured by proximity extension assay (HR, 1.61; *P*=0.03 for eGFR <60; HR, 0.90; *P*=0.72 for eGFR ≥60), with an interaction, *P*=0.04 and 0.11, respectively. No differential pattern was observed when hospitalization for HF was considered (all *P*_interaction_>0.20; Table 2).

**Table 2.**
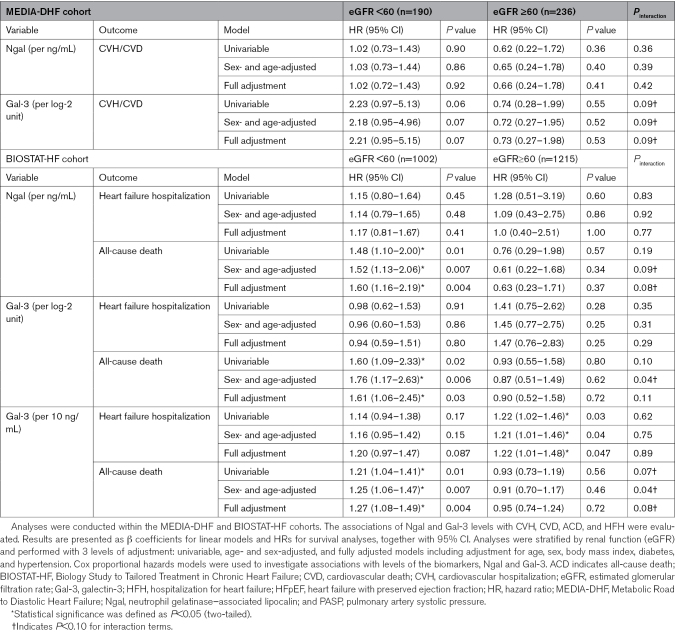
Association of Ngal and Gal-3 Levels With Clinical Outcome According to Renal Function in Patients With HFpEF From the MEDIA-DHF and BIOSTAT-HF Cohorts

These findings, consistent across cohorts and assay platforms, suggest that Ngal is correlated with Gal-3, and the prognostic value of Ngal and Gal-3 for HFpEF is modified by renal function.

## Discussion

Our findings indicate that Ngal inactivation has a cardioprotective effect in the context of CKD in rats. Genetic knockout of *lcn2* and an absence of Ngal are associated with enhanced cardiac perfusion and milder fibrosis, relative to WT littermates with CKD. This protective effect may be partially attributed to a lower level of CFs’ activation. We observed an overexpression of profibrotic and inflammatory genes in CFs following incubation with aldosterone, Ngal, or Gal-3. This effect was attenuated by the MR antagonist finerenone and the inhibitors of Gal-3 and Tlr4. These results suggest a pathway in which MR overactivation leads to increases in Ngal production and Gal-3 levels, with downstream activation of the innate immune response through the Tlr4-Myd88 pathway.

Echocardiography did not highlight changes in systolic parameters, which is expected in CKD models that evolve toward an HFpEF phenotype with preserved systolic function.^23^

### Role of Ngal in the Cardiac Dysfunction and Cardiac Inflammation Associated With CKD

Ngal, also known as lcn2, is a 25-kDa protein from the lipocalin family involved in the innate immune response.^24^ Ngal has emerged as a biomarker of renal injury and cardiovascular stress. It is considered an early biomarker of renal injury as its levels increase markedly within 2 to 4 hours of acute renal injury.^24^ Plasma Ngal levels are also a good indicator of CKD development in patients.^25^ Ngal levels have been associated with an increase in cardiovascular risk.^26,27^ In the Stanislas cohort, serum Ngal levels were positively correlated with systolic pressure and negatively correlated with sodium excretion levels.^28^ High levels of Ngal in patients with HF indicate underlying renal dysfunction and inflammation, which are closely linked to the progression of HF.^29^

The mechanisms underlying the deleterious effects of Ngal in the renal and cardiovascular systems remain unclear. We previously showed that Ngal expression is controlled directly by aldosterone via the MR and mediates MR-induced inflammation and fibrosis in various organs (kidney, heart, and vessels) and cell types (renal distal tubule cells, immune cells, including macrophages in particular, and CFs).^30^ In the immune system, Ngal favors the M1 polarization of macrophages by upregulating the response to interferon gamma and lipopolysaccharide, and suppressing IL-10 signaling.^31^ Ngal deficiency has been shown to decrease the recruitment of neutrophils and macrophages in the heart after ischemia/reperfusion.^31^ We have shown that Ngal is essential for cardiac remodeling and dysfunction after myocardial infarction, via the NF-κB pathway.^13^ We have also shown that Ngal plays a crucial role in aldosterone-induced renal fibrosis and inflammation, by modulating IL-4 signaling in renal macrophages.^32^ Together, these results highlight the potentially detrimental role of Ngal in the cardiac remodeling associated with CKD, as observed here in CKD Ngal knockout rats.

Excess collagen deposition by activated CFs plays a crucial role in increasing cardiac stiffness, disrupting normal heart compliance through the secretion of collagen (types I, III, and VI) and fibronectin, the main components of the ECM (extracellular matrix).^33^ This process results in myocardial stiffening and impaired diastolic function, especially in patients with HFpEF, in whom cardiac stiffness is directly dependent on collagen.^34^ In chronic remodeling scenarios, such as hypertension or postinfarction, sustained fibroblast activation is marked by persistent ECM overproduction.^35^ We, therefore, focused our mechanistic studies on CFs to understand the pathways linking Ngal and cardiac remodeling.

### Gal-3, a Target of Ngal, Is Essential for Ngal Signaling in Rat CFs

Ngal modulated the expression of multiple profibrotic and proinflammatory markers in CFs. We focused on Gal-3, a β-galactoside-binding lectin that we had previously identified as a key mediator of mineralocorticoid-induced cardiac remodeling.^36^ Gal-3 is secreted by activated macrophages and plays a central role in myocardial fibrotic and inflammatory remodeling by promoting myofibroblast proliferation and collagen deposition.^37^ Gal-3 has also emerged as a clinically relevant biomarker in CKD, in which high plasma Gal-3 levels are associated with disease progression.^38^ Studies of patients with HFpEF have reported a correlation between Gal-3 levels and specific diastolic function parameters, such as E/e′ ratio, LV mass, and functional performance metrics, including peak VO_2_ and 6-minute walk distance.^39^ Clinical studies have also reinforced links between plasma Gal-3 concentration and new-onset HFpEF, poor prognosis, and the severity of cardiac structural and functional alterations in HFpEF populations.^40,41^ These findings suggest that Gal-3 promotes inflammation and myocardial fibrosis, thereby contributing to diastolic dysfunction and adverse cardiac remodeling.^41^ Importantly, the pharmacological inhibition of Gal-3 with MCP attenuated the profibrotic and proinflammatory responses induced by rNgal in CFs, suggesting that Gal-3 acts downstream from Ngal to exert its deleterious fibrotic and inflammatory effects.

### Ngal Induces the Tlr4-Myd88 Pathway via Gal-3 in CFs

In our study, we observed a temporal dissociation between gene expression and protein secretion. Specifically, *Col1a1* and *Gal-3* mRNA levels were maximally upregulated at 24 hours, whereas the corresponding secreted proteins (Col1 and Gal-3) displayed significant increases in the culture medium only after 48 hours. This is consistent with the well-established delay between transcriptional activation and extracellular protein accumulation. Similar temporal offsets between mRNA induction and protein secretion have been reported in CF and other cell types,^42,43^ supporting the notion that transcriptional responses precede detectable protein release. Interestingly, rNgal increased both Tlr4 and Myd88 expression, but only Myd88 was reduced by MCP, the Gal-3 inhibitor. This may rely on more stable expression of Tlr4 than Myd88, which is more dynamically regulated and more prone to degradation through proteasomal and autophagy pathways. Such differential regulation helps ensure tight control of Tlr4-mediated signaling and prevents excessive inflammatory activation.^44^

The Tlr4/Myd88 signaling pathway plays a key role in linking innate immune activation to cardiac dysfunction. Upon recognition of pathogen- or damage-associated molecular patterns, Tlr4 recruits Myd88, triggering downstream signaling cascades that result in NF-κB activation and the production of proinflammatory cytokines.^45^ This inflammatory environment promotes CF activation and ECM deposition, processes that impair myocardial compliance.^46^ In mice, deletion or pharmacological inhibition of Myd88 results in reduced fibrosis across multiple organs, and preclinical studies show that knockout of Tlr4 or Myd88 attenuates fibrosis and improves diastolic function in angiotensin II–induced hypertension.^47^ In the context of CKD, sustained activation of the Tlr4/Myd88 axis in cardiac tissue may, therefore, contribute to adverse cardiac remodeling, including hypertrophy and fibrosis. Here, we show that Ngal induces Gal-3, thereby amplifying Tlr4/Myd88 signaling in CFs. Importantly, blockade of this pathway with the Tlr4 antagonist TAK-242 or by Tlr4 silencing demonstrated that Tlr4/Myd88 signaling is essential for the profibrotic and proinflammatory effects of Ngal and its downstream target Gal-3 in CFs.

### Clinical Relevance

The clinical findings reported here are consistent with the Ngal/Gal-3 mechanisms demonstrated in our experimental models. In both settings, high Ngal and Gal-3 levels were clearly associated with high pulmonary artery systolic pressure, a hallmark of advanced diastolic dysfunction, and with adverse outcomes, particularly in patients with impaired renal function. These human data provide a translational validation of our preclinical findings, highlighting the critical role of renal function in modulating the relationship between the Ngal/Gal-3 axis, and the severity and prognosis of HFpEF. Supporting evidence from other clinical studies further reinforces this link. In patients with diabetes, plasma Ngal levels are independently associated with LV hypertrophy, even after adjusting for age, sex, body mass index, eGFR, and systolic blood pressure.^48^ A recent meta-analysis also found that plasma Gal-3 serves as a predictive biomarker for new-onset HFpEF, adverse prognosis in established HFpEF, and the severity of LV diastolic dysfunction. Notably, in patients with HF with reduced ejection fraction, Ngal levels are independently correlated with Gal-3 levels, even after adjusting for potential confounders such as age, ejection fraction, and eGFR.^40^

This underscores the therapeutic potential of targeting this pathway, especially in patients with concomitant HFpEF and renal dysfunction, a subgroup at markedly high risk for poor clinical outcomes.

### Limits of the Study

This study relied on the 5/6 nephrectomy model to induce CKD to evaluate its effects on cardiac structure and function while exploring the mechanistic role of Ngal in the absence of a primary cardiac insult. However, this model represents an abrupt loss of renal mass and may not fully reflect the progressive nature of human CKD. Future studies would benefit from evaluating alternative CKD models characterized by more gradual renal dysfunction, including those associated with metabolic disease. In addition, our preclinical experiments were conducted exclusively in male rats, which are known to develop more pronounced renal dysfunction and fibrosis in experimental CKD models.^49^ In contrast, the BIOSTATS-HF and MEDIA-DHF cohorts included both men and women, ensuring that our findings remain relevant and applicable to both sexes in clinical practice.

### Conclusions

Our results show that inactivation of the *Ngal* gene in rats prevents impairment of LV perfusion and interstitial fibrosis in the context of CKD. We found that Ngal activates fibrotic and inflammatory pathways via Gal-3 and the Tlr4-Myd88 axis in CFs. Targeting Ngal is, therefore, a potential novel therapeutic approach for limiting cardiac remodeling in CKD.

### Perspectives

Ngal has emerged as a biomarker of renal injury. Our data suggest that targeting Ngal may be of interest for limiting cardiovascular outcomes in CKD. Ngal may, therefore, represent a novel therapeutic target in this and potentially other contexts.

## Article Information

### Acknowledgments

The authors thank the Phenomin-Institut Clinique de la Souris for expert assistance in establishing the lipocalin-2/Ngal (neutrophil gelatinase–associated lipocalin) mutant rat line. The authors are also grateful for the excellent technical assistance and support for animal care provided by the center d’Exploration Fonctionelles. The graphic abstract was created with BioRender.com.

### Author Contributions

F. Jaisser and N. Lopez-Andres provided the concept and design of research. M. Soulié, T. Sánchez-Bayuela, I. Lima-Posada, L. Nicol, Y. Stephan, and P. Mulder performed experiments and prepared figures. M. Soulié, T. Sánchez-Bayuela, P. Mulder, N. Lopez-Andres, N. Girerd, and F. Jaisser analyzed data and interpreted the results of experiments. F. Jaisser, N. Girerd, A. Voors, J. Lagrange, and M. Soulié drafted the manuscript. M. Soulié, T. Sánchez-Bayuela, I. Lima-Posada, Y. Stephan, L. Nicol, P. Mulder, N. Lopez-Andres, N. Girerd, and F. Jaisser approved the final version of the manuscript.

### Disclosures

F. Jaisser received honoraria and research grants from AstraZeneca and Bayer. The other authors report no conflicts.

## Supplementary Material
