# Supplementary material for: Neutrophil Gelatinase–Associated Lipocalin Drives Cardiac Remodeling in Rats With Chronic Kidney Disease
Source: Hypertension. 2026 Feb 17;83(6):e25658. doi: 10.1161/HYPERTENSIONAHA.125.25658 (PMC13189387; doi:10.1161/HYPERTENSIONAHA.125.25658)
Supplement: Supplementary file 1 [file hyp-83-e25658-s001.pdf]

SUPPLEMENTAL MATERIAL

ARTICLE: Neutrophil Gelatinase-Associated Lipocalin drives cardiac remodeling in rats with chronic kidney disease

Matthieu Soulié[1,2]*, Tania Sánchez-Bayuela[1]*, Ixchel Lima-Posada[1], Yohan Stephan[2], Lionel Nicol[2], Zohra Lamiral[3], Jeremy. Lagrange[3], Adriaan Voors[4], Natalia Lopez-Andres[5], Nicolas Girerd[3], Paul Mulder[2], and Frédéric Jaisser[1,3]

(1) UMR ICAN 1166, Sorbonne Université, Faculty of Medicine, Institute of Cardiometabolism and Nutrition (ICAN), Paris, France.

(2) Normandie Univ, UNIROUEN, INSERM U1096, Rouen, France

(3) Université de Lorraine, INSERM Centre d'Investigations Cliniques-Plurithématique 1433, UMR 1116, CHRU de Nancy, French-Clinical Research Infrastructure Network (F-CRIN) INI-CRCT, Nancy, France

(4) University Medical Centre Groningen, Department of Cardiology, University of Groningen, Hanzeplein 1, 9713 GZ, Groningen, The Netherlands

(5) Cardiovascular Translational Research. Navarrabiomed (Miguel Servet Foundation), Instituto de Investigación Sanitaria de Navarra (IdiSNA), Hospital Universitario de Navarra (HUN), Universidad Pública de Navarra (UPNA), Pamplona, Spain

* Equal contributions

Short title: Ngal and CKD-associated cardiac remodeling

Correspondence to: frederic.jaisser@inserm.fr;

ORCID ID: 0000-0001-9051-1901

Supplemental Methods

Knockout ngal rat generation

The rat Ngal mutant line was generated at the Institut Clinique de la Souris – PHENOMIN-ICS. To generate a targeted deletion of the Lcn2 genomic region encompassing exons 2 to 5 (ENSRNOE00000133390 to ENSRNOE00000133734; approximately 1.8 kb), a CRISPR/Cas9 strategy was employed. Specifically, the region was excised using two pairs of small guide RNAs (sgRNAs): the 5' sgRNAs (CTGCCCTGGAACTGCAAGAA [gR68] and TCGGCCTGTG366AGGCCACCCC [gR74]) and the 3' sgRNAs (CTGTCTTGTATTGACCT366ACC [gR88] and AAGTCTTCCCTTCCCCTGGT [gR67]). The sgRNA identifiers correspond to the MIT specificity scores as determined by the CRISPOR web tool. Sprague Dawley oocytes were microinjected with a mixture of the four sgRNAs (12 ng/µl each) and spCas9 mRNA (25 ng/µl). Germline transmission was achieved by breeding three founders, resulting in the establishment of the mutant line. Genotyping was performed by PCR. For deletion detection, primers F1 (CTGTCAGGTAGGAATCTTTTCAGTC) and R8 (AGCTT366GGACTTGAACATGCGATCTT) yielded a 440 bp product. For WT detection, F1 and R1 (ATGACCCGGTTCTGGGCTGTAACAG) produced a 445 bp product. Heterozygous offspring were intercrossed to obtain homozygous mutants.

Echocardiography

Echocardiography was performed one week before end of the protocol. Rats were anesthetized with isoflurane (ISO-VET®, 2.5% induction, 1.5% maintenance), and scans were done using a Vivid 7 with an M12L probe. A short-axis view at the papillary muscles was used to obtain M-mode tracings, and LV diameters measured per American Society of Echocardiology method. LV outflow velocity was assessed by pulsed-wave Doppler, and cardiac output calculated as $CO = \text{aortic VTI} \times [\pi \times (\text{LV outflow diameter}/2)^2] \times \text{heart rate}$. Echocardiography was performed blinded on the same day.

Biochemical studies

Twenty-four-hour urine collection was performed for all studied groups 10 weeks after the last surgery using metabolic cages. Plasma urea and creatinine levels, as well as urinary creatinine levels, were determined using an automatic analyzer (Konelab 20i; Thermo Fisher Scientific, Vernon Hills, IL, United States). Albuminuria has been measured using an Albumin ELISA Kit at the end of the protocol (Crystal Chem, Elk Grove Village, IL, United States #80630).

Myocardial perfusion

The last week before the end of the protocol myocardial perfusion was measured in methohexital (50 mg/kg IP, Hikma Pharmaceuticals PLC) anesthetized animals using a Bruker Biospec 4.7 Tesla MRI. T1 sequence acquisition was done without contrast agent.

Glomerular filtration rate

Glomerular filtration rate (GFR) was assessed by intravenous injection of fluorescein isothiocyanate (FITC)-sinistrin into the lateral caudal vein. FITC-sinistrin clearance was monitored transcutaneously using a Mini GFR Monitor (MediBeacon, St. Louis, MO) positioned on the shaved dorsal skin. Data acquisition and GFR calculations were performed with MB_Studio2 software (MediBeacon)[50].

Histology

The heart and kidneys were harvested at the end of the study. Tissues were fixed in 4% PFA at 4 °C overnight, embedded in paraffin, and sectioned at 7 µm using a microtome. Sirius red staining was used to determine interstitial fibrosis and images were taken in polarized light, Renal and LV (septum and LV free wall) fibrosis was calculated as the percentage of the area containing collagen to the total area of the image of the staining. Images were obtained with lame scanner Axio Scan.Z1, Zeiss (ZEN 3.1 Zeiss software). All analyses were blinded.

Cell culture and treatments

Rat primary cardiac fibroblasts (CFs) (P10402, Innoprot, Spain) isolated from adult Sprague Dawley rat heart tissue were cultured according to the manufacturer's instructions in cardiac fibroblast medium supplemented with fibroblast growth factors (P60108, Innoprot, Spain), 10% FBS, and 1% penicillin/streptomycin. Prior to stimulation, cells were maintained in low-serum medium (2% FBS) and then treated with aldosterone (Aldosterone; MR agonist, 10^{-8} M), rat rNgal (500 ng/mL; R&D Systems), or rGal-3 (5 µg/mL; Abbexa, France), in the absence or presence of either Modified Citrus Pectin (MCP; Gal-3 inhibitor, 500 µg/mL), TAK-242 (TLR4 inhibitor, 1 µM; MedChem, France), or Finerenone (Fine; MR antagonist, 10^{-6} M).The concentrations of Aldosterone, MCP, TAK-242, and Fine were selected based on previous studies[51-54]. The concentrations and time points for rGal-3 (10^{-8} M; 5 µg/mL) and rNgal (500 ng/mL) were determined by dose–response and time-course experiments (Fig. S4).

Protein and gene expression analyses

Secreted protein levels were quantified by ELISA (R&D Systems, DY3144, DY506, DY6220, DY350, France) at 48h, selected as the optimal time point for protein secretion (Fig. S4). Intracellular protein levels were measured by Western blot (WB) at 24h. Cells were lysed using cOmplete Lysis-M reagent (Roche, Germany). Gene expression was assessed by RT–qPCR at 24h, based on time-course experiments (Fig. S4). Total RNA was extracted using a TRIzol-based method, and reverse transcription was performed using M-MLV reverse transcriptase (Thermo Fisher Scientific, USA) according to the manufacturer's instructions. Q-PCr was performed using iQ SYBR Green Supermix (Bio-Rad, USA). Gene expression was calculated using the $2^{-\Delta\Delta Ct}$ method and normalized to *Gapdh* as the reference gene. Intracellular protein levels determined by WB were normalized to total protein content using Criterion TGX Stain-Free Gels (Bio-Rad). A complete list of the antibodies and primers used is provided in Table S2.

Measurement of NFκB activity

To assess the activivity of the nuclear transcription factor NFκB, the commercial TransAM™ NFκB p65 Transcription Factor Assay (Active Motif) was used. This assay is based on the ability of active NFκB to bind to the consensus sequence 5′-GGGACTTTCC-3′, which is immobilized on an ELISA-type plate. NFκB present in cell extracts specifically binds to this oligonucleotide. The primary antibodies used for detection recognize epitopes on the p65 subunits, which are accessible only when NFκB is activated and bound to its target DNA. Detection is performed using an HRP-conjugated secondary antibody, providing a sensitive colorimetric signal that can be quantified spectrophotometrically. For this experiment, CF were incubated for 24h with the required stimuli (Ngal/Gal3) ± the Gal3 inhibitor MCP at the concentrations previously detailed. Protein extraction and subsequent analysis were performed according to the manufacturer's instructions.

Experiments using small-interfering RNA (siRNA)

CF were seeded into 12-well plates and cultured in complete fibroblast medium (Innoprot) containing 10% FBS, 2% antibiotic–antimycotic, and fibroblast supplements until reaching approximately 80% confluence. Cells were then serum-reduced (2% FBS) for 24 h prior to transfection. Gene silencing of Tlr4 was performed using small interfering (si)-RNAs (Genecust, France), provided as a pool of four distinct sequences to increase knockdown efficiency. Negative control siRNA was included in parallel. Transfections were carried out at a final siRNA concentration of 50 nM using a lipid-based approach with Lipofectamine (Invitrogen) in Opti-MEM medium (Gibco Invitrogen, Carlsbad, CA). CF were incubated with the transfection mixture for 6 h, after which activation medium was added for an additional 24 h. Following this incubation period, cells were stimulated for 24-48h with rNGAL or rGal3 as described. The sequences of the primers were as follows: Tlr4; Pair 1: F: GGCUCAUAAUCUUAUACAUTT//R: AUGUAUAAGAUUAUGAGCCTT, Pair 2: CUGGAUCCAUCCCAUUAUATT//R: UAUAAUGGGAUGGAUCCAGTT, Pair3: CGAGCUGGUAAAGAAUUUATT//R: UAAAUUCUUUACCAGCUCGTT, Pair 4: GGCUGGCAAUUCUUUCAAATT// R: UUUGAAAGAAUUGCCAGCCTT. Negative control: F: UUCUCCGAACGUGUCACGUTT// R: ACGUGACACGUUCGGAGAATT.

Supplemental references

50. Scarfe L, Schock-Kusch D, Ressel L, Friedemann J, Shulhevich Y, Murray P, Wilm B, de Caestecker M. Transdermal Measurement of Glomerular Filtration Rate in Mice. *J Vis Exp*. 2018;58520.

51. Martínez-Martínez E, Calvier L, Fernández-Celis A, Rousseau E, Jurado-López R, Rossoni LV, Jaisser F, Zannad F, Rossignol P, Cachofeiro V, et al. Galectin-3 blockade inhibits cardiac inflammation and fibrosis in experimental hyperaldosteronism and hypertension. *Hypertension*. 2015;66:767–775.

52. Calvier L, Miana M, Reboul P, Cachofeiro V, Martinez-Martinez E, de Boer RA, Poirier F, Lacolley P, Zannad F, Rossignol P, et al. Galectin-3 mediates aldosterone-induced vascular fibrosis. *Arterioscler Thromb Vasc Biol*. 2013;33:67–75.

53. Martínez-Martínez E, Buonafine M, Boukhalfa I, Ibarrola J, Fernández-Celis A, Kolkhof P, Rossignol P, Girerd N, Mulder P, López-Andrés N, et al. Aldosterone Target NGAL (Neutrophil Gelatinase-Associated Lipocalin) Is Involved in Cardiac Remodeling After Myocardial Infarction Through NFκB Pathway. *Hypertension*. 2017;70:1148–1156.

54. Ono Y, Maejima Y, Saito M, Sakamoto K, Horita S, Shimomura K, Inoue S, Kotani J. TAK-242, a specific inhibitor of Toll-like receptor 4 signalling, prevents endotoxemia-induced skeletal muscle wasting in mice. *Sci Rep*. 2020;10:694.

Fig. S1 In vivo experimental design and Ngal knockout validation. A experimental design, **B** cardiac *Ngal* protein expression. Transcript levels were normalized to *Gapdh* and expressed relative to control. Data are shown as mean ± SEM, n = 4-9. Statistical analysis was performed using one-way ANOVA followed by Tukey's post hoc test. *p < 0.05, **p < 0.01, **p < 0.001.

Figure legends

Fig. S1 In vivo experimental design and Ngal knockout validation.

A experimental design, **B** cardiac Ngal protein expression. Transcript levels were normalized to Gapdh and expressed relative to control. Data are shown as mean ± SEM, n = 4-9. Statistical analysis was performed using one-way ANOVA followed by Tukey's post hoc test. *p < 0.05, **p < 0.01, **p < 0.001.

Fig. S2. Ngal knockout does not modify kidney dysfunction in rats with chronic kidney disease.

CKD: chronic kidney disease. **A** Urea (mM), **B** creatinine (µM), **C** Albuminuria (mg/24h), **D** kidney weight (g), **E** GFR (ml/min/100g body weight), **F** Fibrosis (%). Data are shown as mean ± SEM, n = 8-10. Statistical analysis was performed using one-way ANOVA followed by Tukey's post hoc test. *p < 0.05, **p < 0.01, **p < 0.001.

Fig. S3. NGAL knockout reduces MyD88 expression without altering TLR4 or Galectin-3 protein levels

CKD: chronic kidney disease. **A** Protein levels of Tlr4, **B** Myd88 measured by western blot and **C** Galectine 3 (Gal-3) by ELISA. B Data are shown as mean ± SEM, n = 6 biological replicates. Statistical analysis was performed using student t test. *p < 0.05.

Fig. S4. Dose-response and time course of CFs treated with rNgal and rGal-3

Relative expression (Δ ΔCt values) of key target genes (Col1a1, Ccl2 and Il6) of rNgal and rGal-3 at different doses and time points. **A**, CFs treated with different doses of rNgal (500 ng/mL, 50 ng/mL and 5 ng/mL) for 24h. **B**, CFs treated with different doses of rGal-3 (10^{-8}M, 5 µg/mL and 10^{-7}M, 50 µg/mL) for 24h. **C**, CFs treated with rNgal

(500 ng/mL) for 24/48h. **D**. CFs treated with rGal-3 (10^{-8}M, 5 µg/mL) for 24/48h. Transcript levels were normalized to *Gapdh* and expressed relative to control. Data are shown as mean ± SEM, n = 5–6 biological replicates. Statistical analysis was performed using one-way ANOVA followed by Tukey's post hoc test. *p < 0.05, **p < 0.01, **p < 0.001.

Fig. S5. Aldosterone/Mineralocorticoid receptor signaling drives fibrotic and inflammatory responses in CFs. Relative expression (Δ Δ Ct values) of fibrotic markers in cardiac fibroblasts (CFs) treated or not with Aldosterone 10^{-8M} for 24h measured by qPCR. **A** *Col1a1*, collagen type 1; **B** *Col3a1*, collagen type 3; **C** *Ccl2*/Mmp1, Chemokine (C–C motif) Ligand 2; **D** *IL-6*, interleukin-6, **E** *Ngal*, Neutrophil-gelatinase associated lipocalin and **F** *Gal-3*, galectin-3. Target transcript expression was normalized to *Gapdh* and presented relative to control. Data are shown as mean ± SEM, n = 5–6 biological replicates. Statistical analysis was performed using one-way ANOVA followed by Tukey's post hoc test. *p < 0.05, **p < 0.01, **p < 0.001

Fig. S6. The expression of TLRs signaling through MyD88 in CFs.

Relative expression (Δ Δ Ct values) of Toll-like Receptors (TLRs) cardiac fibroblasts (CFs). **A** Tlrs expressed in basal CFs. **B**, Tlrs expression in CFs treated or not with rNGAL (500 ng/mL)/ rGal-3 (5 µg/mL) ± Modified Citrus Pectin (MCP, 500 µg/mL) for 24h measured by qPCR. Target transcript expression was normalized to *Gapdh* and presented relative to control. Data are shown as mean ± SEM, n = 5–6 biological replicates. Statistical analysis was performed using one-way ANOVA followed by Tukey's post hoc test. *p < 0.05, **p < 0.01, **p < 0.001.

Fig. S7. Inhibition of Tlr4 prevents the induction of inflammatory and fibrotic gene expression by rGal-3/rNgal in CFs.

CFs were treated for 24 h with rNgal (500 ng/mL) or rGal-3 (5 µg/mL) in the presence or absence of TAK-242 (1 µM, TLR4 inhibitor), and gene expression was quantified. **A** Expression of Tlr4 (Toll-like receptor 4), Myd88 (myeloid differentiation primary response 88), Irak1 (interleukin-1 receptor–associated kinase 1) and Irak4 (interleukin-1 receptor–associated kinase 4). **B** Expression of fibrotic markers Col1a1 (collagen type I alpha 1) and Col3a1 (collagen type III alpha 1), and inflammatory markers Ccl2 (C-C motif chemokine ligand 2) and Il6 (interleukin-6). Target gene expression was normalized to *Gapdh* and expressed relative to untreated control. Data are shown as mean ± SEM, n = 5–6 biological replicates. Statistical analysis was performed using one-way ANOVA followed by Tukey's post hoc test. *p < 0.05, **p < 0.01, ***p < 0.001.

Fig. S8. Silencing of *Tlr4* prevents the induction of inflammatory and fibrotic gene expression by rGal-3/rNgal in CFs.

CFs were treated for 24h with rNgal (500 ng/mL) or rGal-3 (5 µg/mL) in the presence or absence of small interfering RNA (si-RNA) , and gene expression was quantified. **A**

Tlr4 gene measurement in cells treated with the si-*Tlr4* or si-Ctrl. **B** Expression of Tlr4 (Toll-like receptor 4), Myd88 (myeloid differentiation primary response 88), Irak1 (interleukin-1 receptor–associated kinase 1) and Irak4 (interleukin-1 receptor–associated kinase 4). **C** Expression of fibrotic markers Col1a1 (collagen type I alpha 1) and Col3a1 (collagen type III alpha 1), and inflammatory markers Ccl2 (C-C motif chemokine ligand 2) and Il6 (interleukin-6). Target gene expression was normalized to *Gapdh* and expressed relative to untreated control. Data are shown as mean ± SEM, n = 5–6 biological replicates. Statistical analysis was performed using one-way ANOVA followed by Tukey's post hoc test. *p < 0.05, **p < 0.01, ***p < 0.001.

Table legends

Table S1. **Ngal knockout effects on cardiac parameters and body weight in rats with chronic kidney disease model.** CKD: chronic kidney disease. LVEDD: left ventricle end diastolic diameter (mm), LVESD: left ventricle end systolic diameter (mm), fractional shortening (%), stroke volume (ml), cardiac output (ml/min), left ventricle weight (g), body weight (g), Data are shown as mean ± SEM; n = 8-10. Statistical analysis was performed using one-way ANOVA followed by Tukey's post hoc test. *p < 0.05. vs Sham Ngal knockout

Table S2. **List of antibodies and primers used for Western blot and qPCR.**

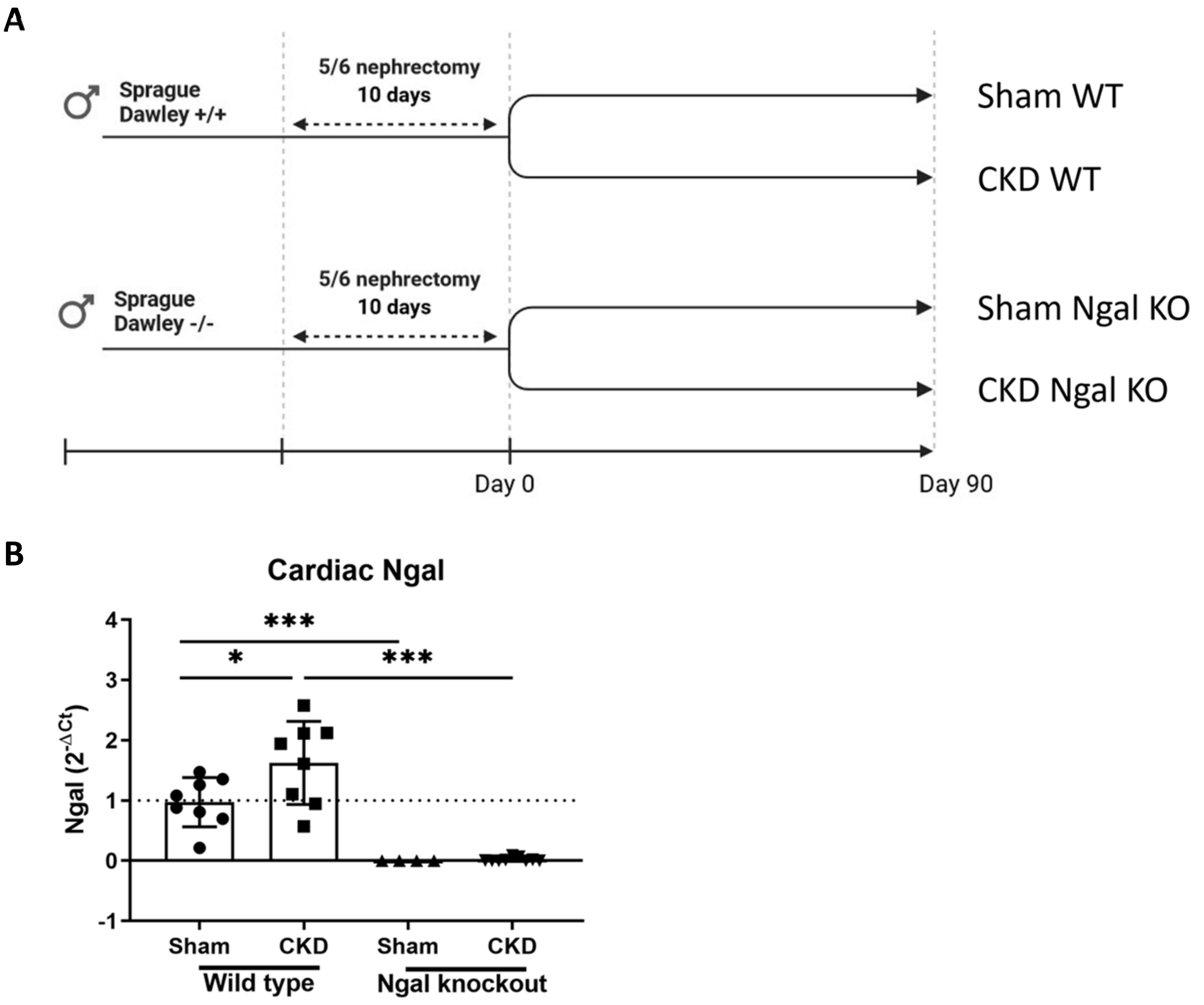

Fig. S1 In vivo experimental design and Ngal knockout validation.

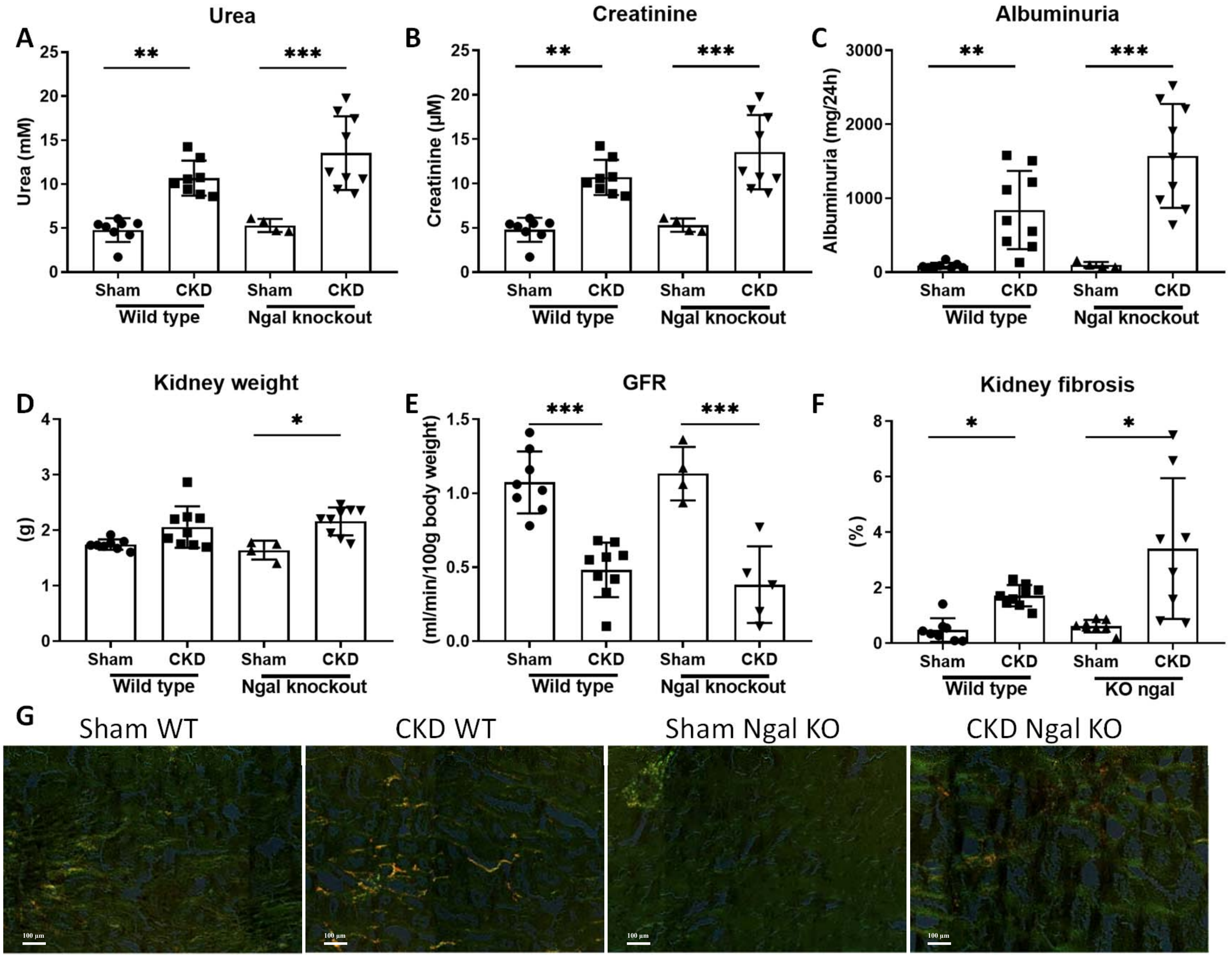

Fig. S2. **Ngal knockout does not modify kidney dysfunction in rats with chronic kidney disease.**

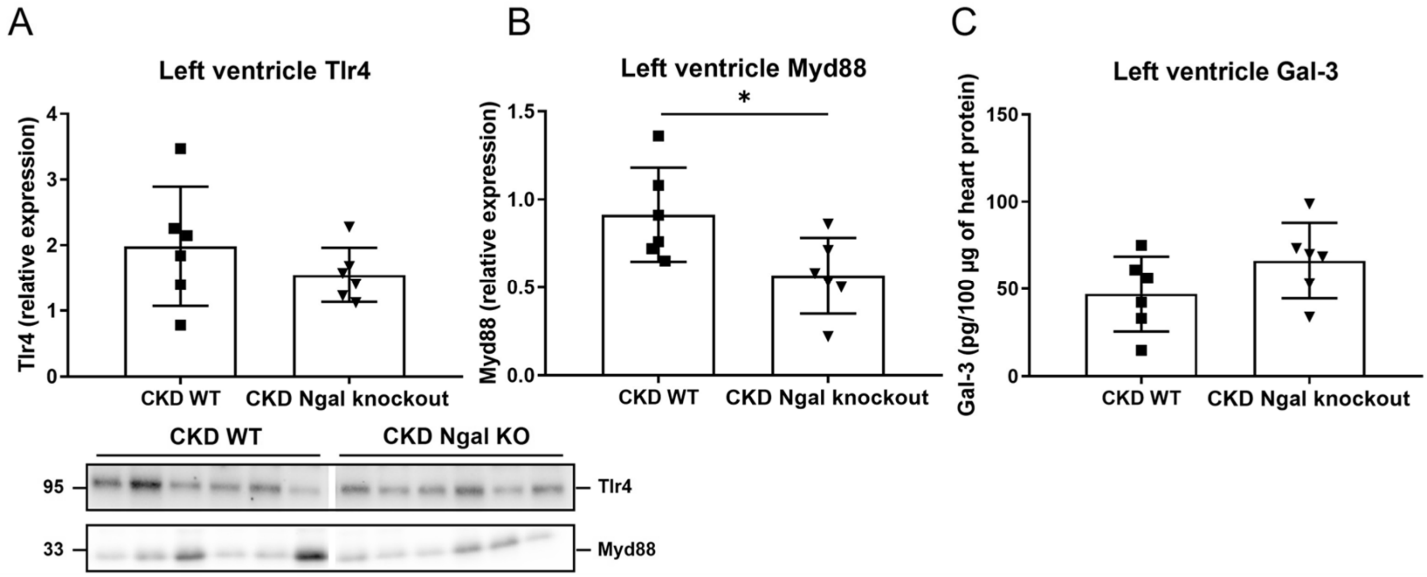

Fig. S3. **NGAL knockout reduces MyD88 expression without altering TLR4 or Galectin-3 protein levels**

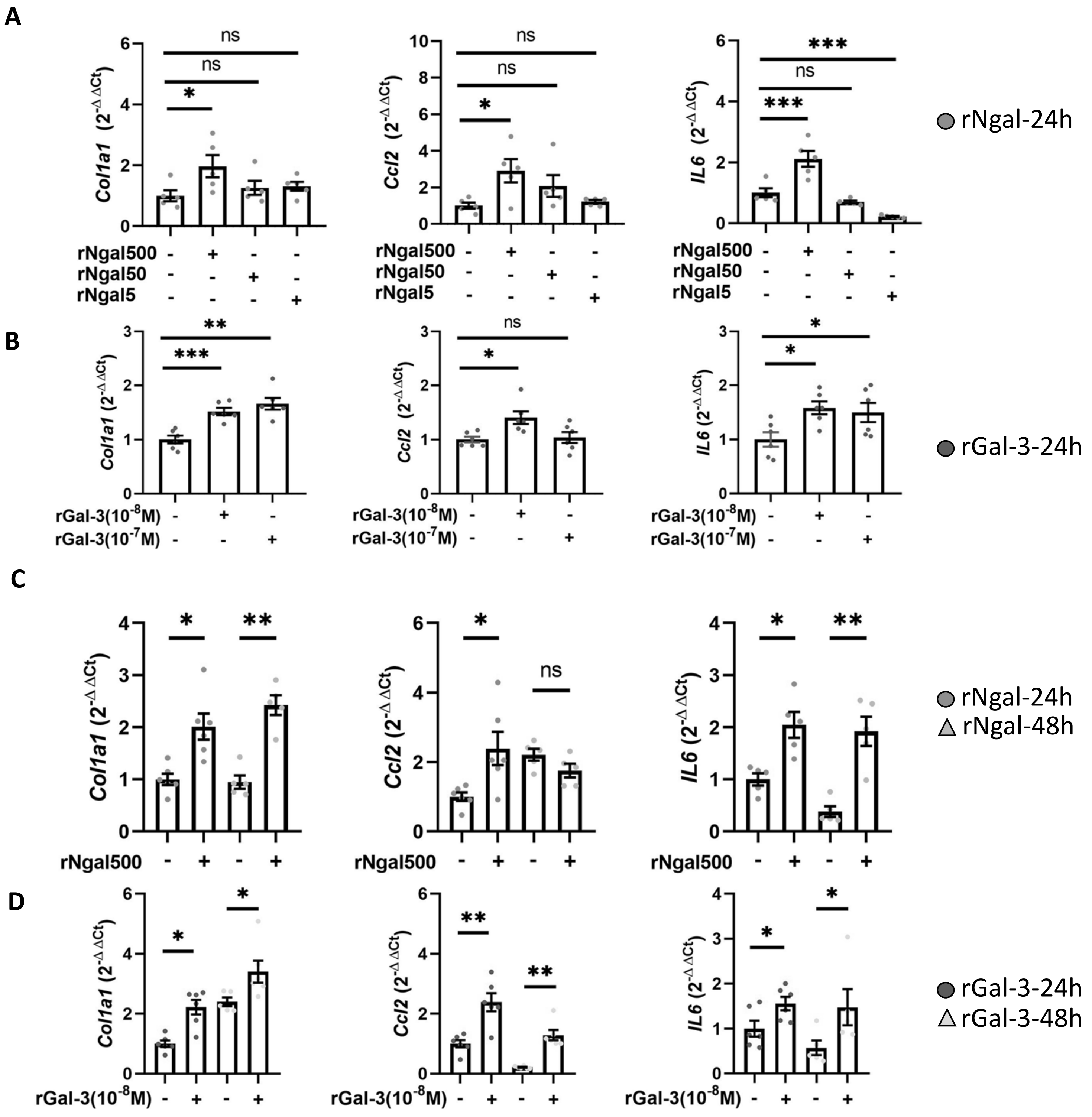

Fig. S4. Dose-response and time course of CFs treated with rNgal and rGal-3

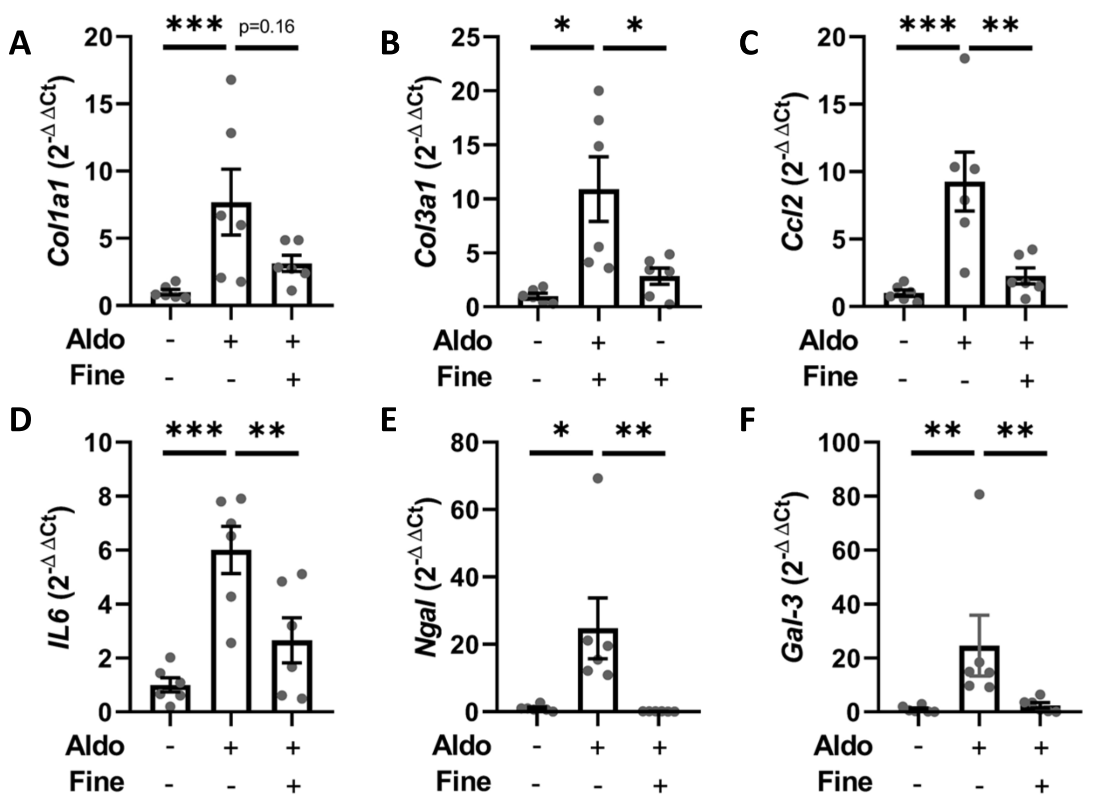

Fig. S5. Aldosterone/Mineralocorticoid receptor signaling drives fibrotic and inflammatory responses in CFs.

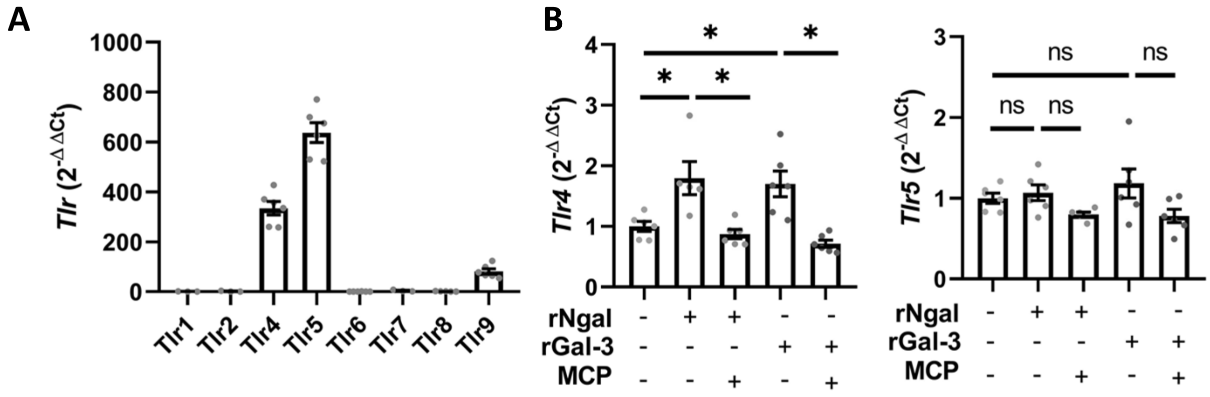

Fig. S6. The expression of TLRs signaling through MyD88 in CFs.

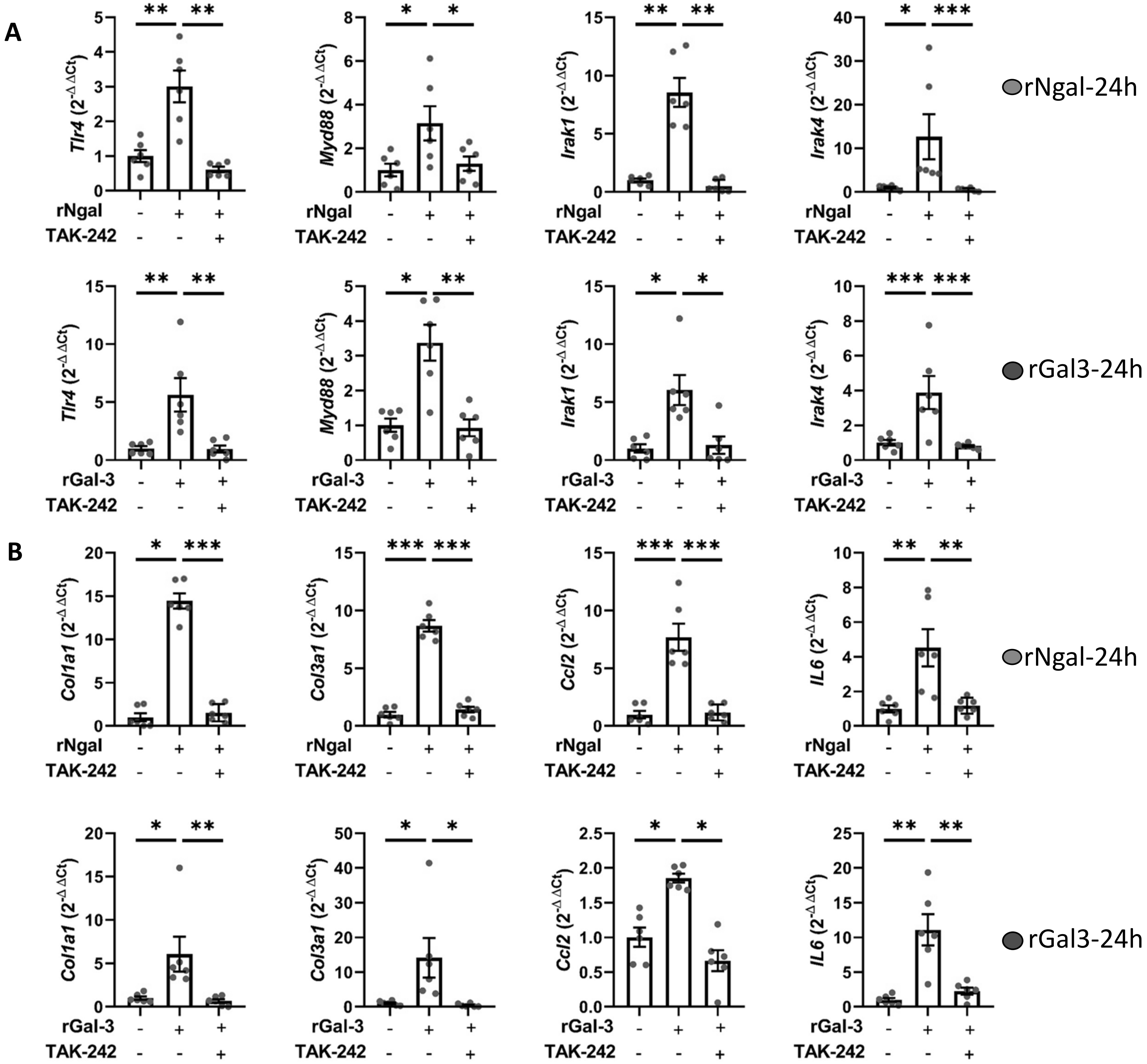

Fig. S7. Inhibition of Tlr4 prevents the induction of inflammatory and fibrotic gene expression by rGal-3/rNgal in CFs.

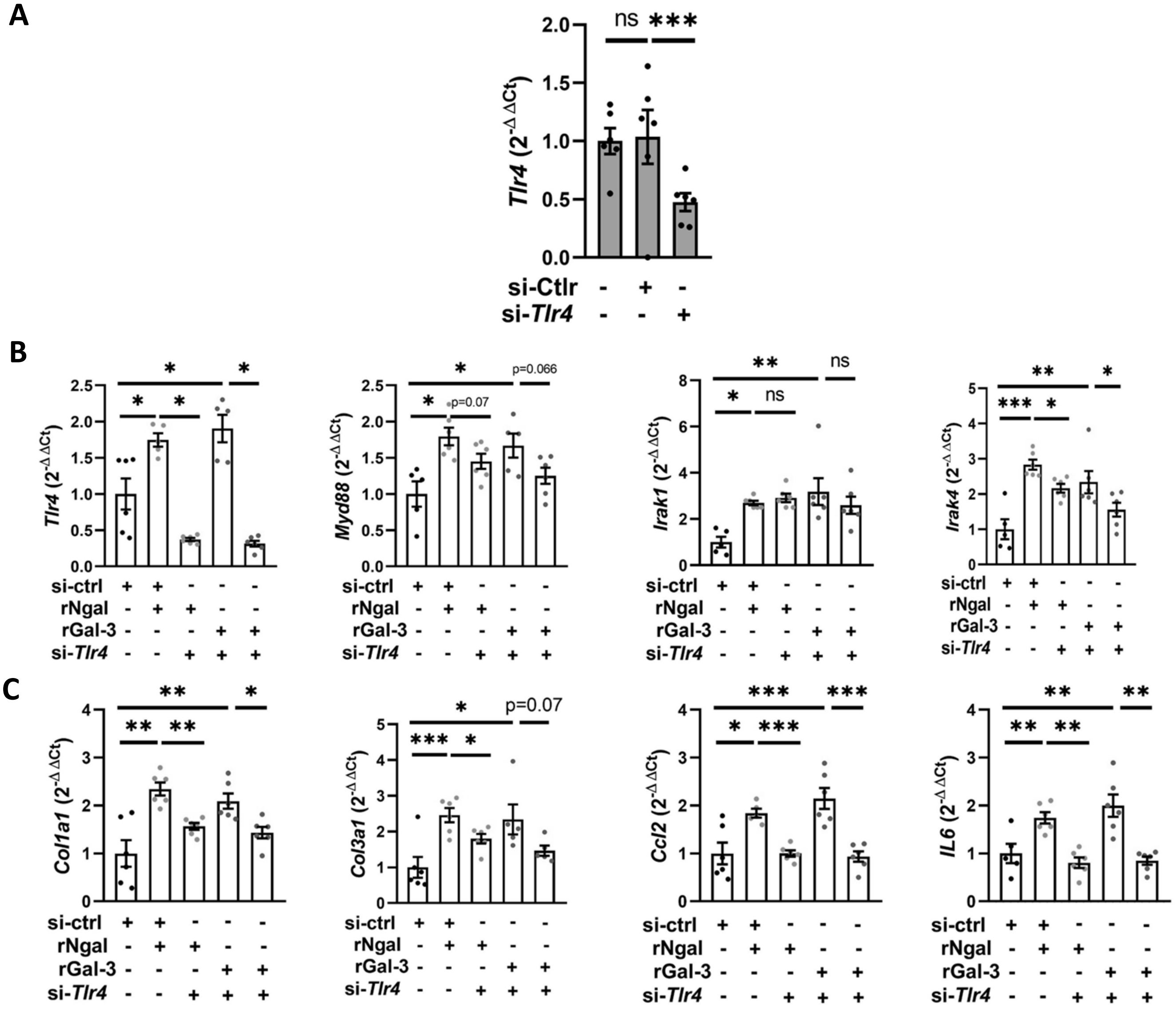

Fig. S8. Silencing of *Tlr4* prevents the induction of inflammatory and fibrotic gene expression by rGal-3/rNgal in CFs.

Group	WT		Ngal KO	
	Sham	CKD	Sham	CKD
LVEDD (mm)	7.61±0.15	7.72±0.24	7.95±0.33	7.64±0.22
LVESD (mm)	3.56±0.12	3.83±0.27	4.27±0.12	4.40±0.17
Fractional shortening (%)	53.3±1.2	51.0±1.9	46.3±1.6	42.8±0.8*
Stroke volume (ml)	0.41±0.01	0.40±0.01	0.41±0.02	0.39±0.01
Cardiac output (ml/min)	141±4	130±3	140±4	130±5
Left ventricle weight (g)	1.03±0.03	0.97±0.03	0.94±0.03	1.04±0.04
Body weight (g)	599±14	562±17	567±17	532±11

Table S1. Ngal knockout effects on cardiac parameters and body weight in rats with chronic kidney disease model.

List of antibodies used for Western Blot.

Target antigen	Supplier	Catalog#	Working dilution	Target antigen
TLR4	Santa Cruz	Sc-293072	1:500	TLR4
MyD88	Cell signaling	4283s	1:1000	MyD88
Goat anti-rabbit IgG (H+L)	Invitrogen	31480	1:10000	Goat anti-rabbit IgG (H+L)
Goat anti-mouse	Thermoscientific	31430	1:5000	Goat anti-mouse

List of primers used for the real time qPCR

Gene	Forward sequence (5'-3')	Reverse sequence (5'-3')
Ccl2	TTCCTTATTGGGGTCAGCAC	CAGTTAATGCCCCACTCACC
Col1a1	GCCTCCCAGAACATCACCTA	ATGTCTGTCTTGCCCCAAGT
Gapdh	GTGGCAAAGTGGAGATTGTTGCC	GATGATGACCCtTTTGGCTCC
Il6	AACTCCATCTGCCCTTCAGGAACA	AAGGCAGTGGCTGTCAACAACATC
Irak1	TGAAGGAGGAGGCTGACCTA	GGGGCTGTCCTGATGTAAAA
Irak4	GACTTGCGGCTGTGGATG	TCTCGTGCAGACACTGGCTA
Myd88	GACAAAGGAACTGGGAGGCAT	CCGGATCATCTCCTGCACAAA
Tlr4	TCQACAACTTCAGTGGCTGGAT	TGTCAGTACGGTTGAGAGC
Ngal	CGATGAACTGAAGGAGCGAT	TCTGGCAACAGGAAAGATGG
Gal3	AGCCCAACGCAAACAGTATC	AGCCCAACGCAAACAGTATC

Table S2. **List of antibodies and primers used for Western blot and qPCR.**
