# Supplementary figures and images for: Neutrophil Gelatinase–Associated Lipocalin Drives Cardiac Remodeling in Rats With Chronic Kidney Disease
Source: Hypertension. 2026 Feb 17;83(6):e25658. doi: 10.1161/HYPERTENSIONAHA.125.25658 (PMC13189387; doi:10.1161/HYPERTENSIONAHA.125.25658)

## Cardiac fibroblasts

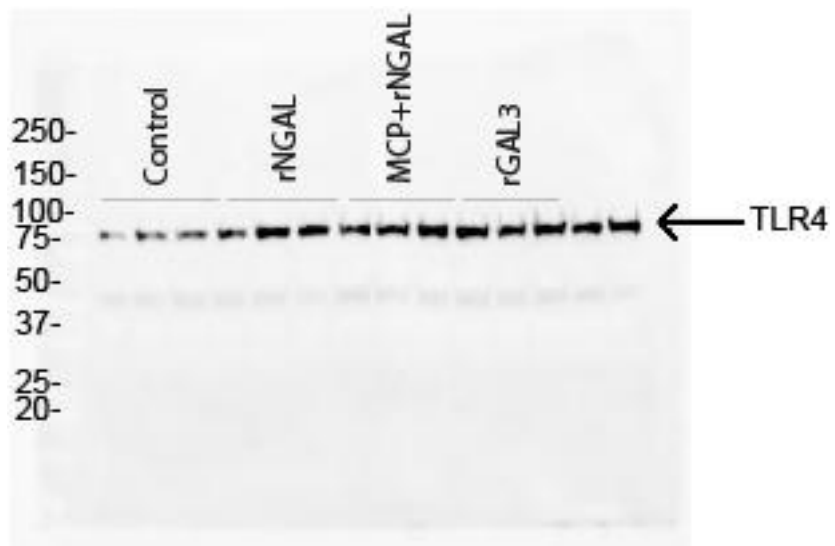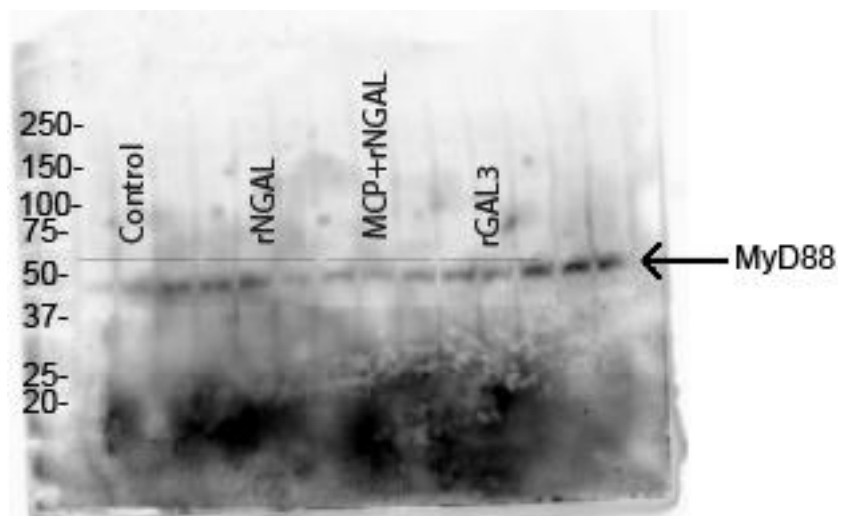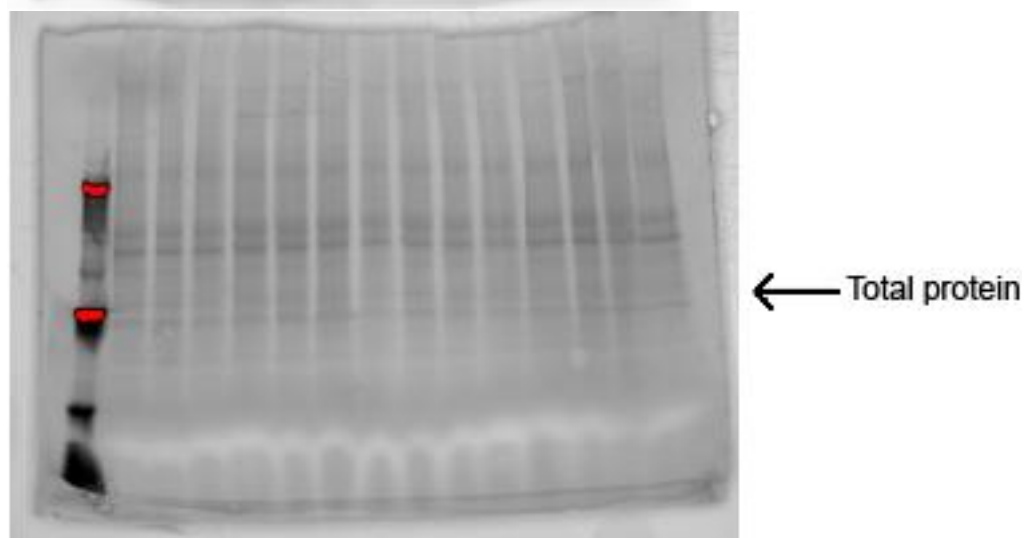

In Vivo

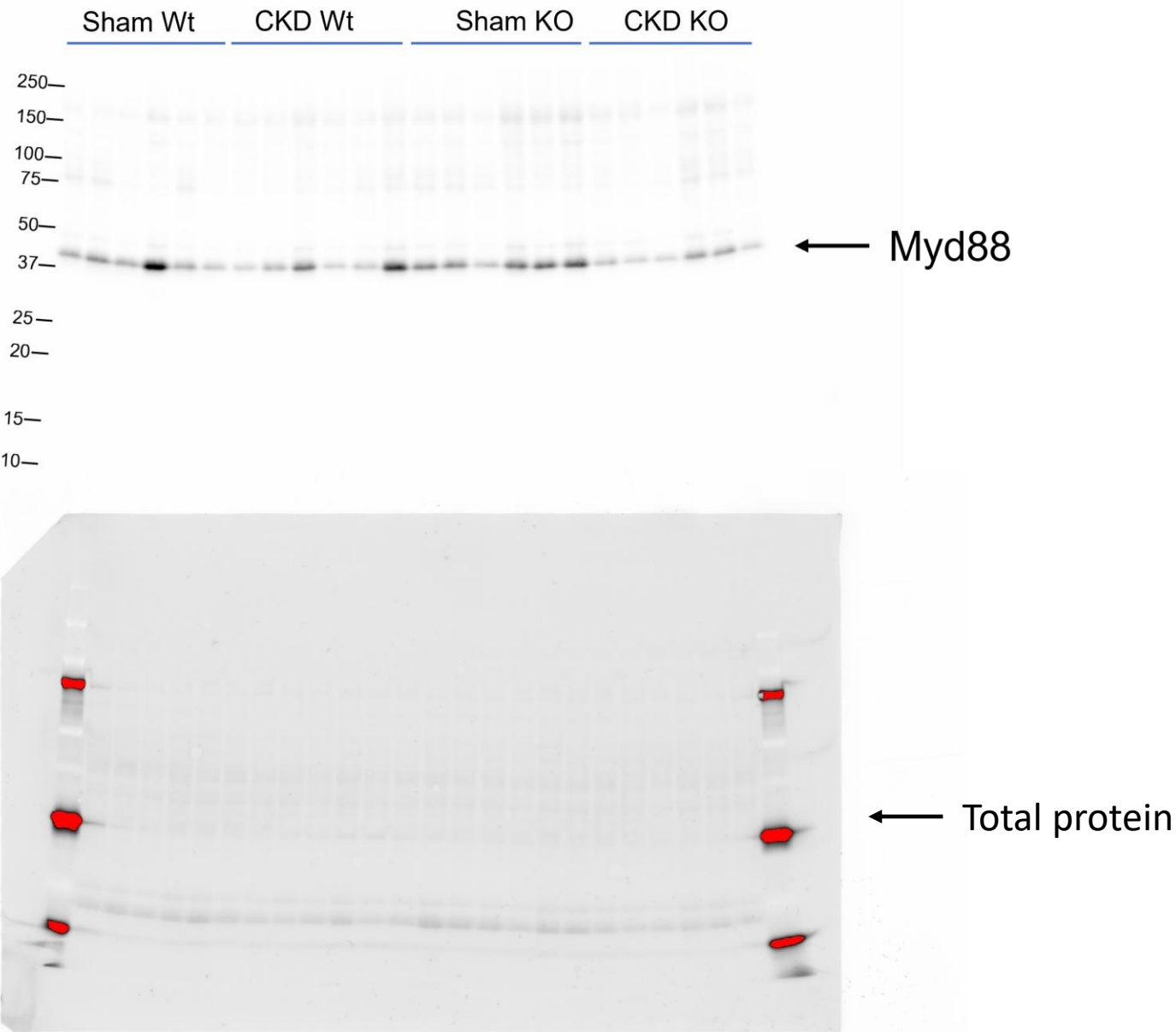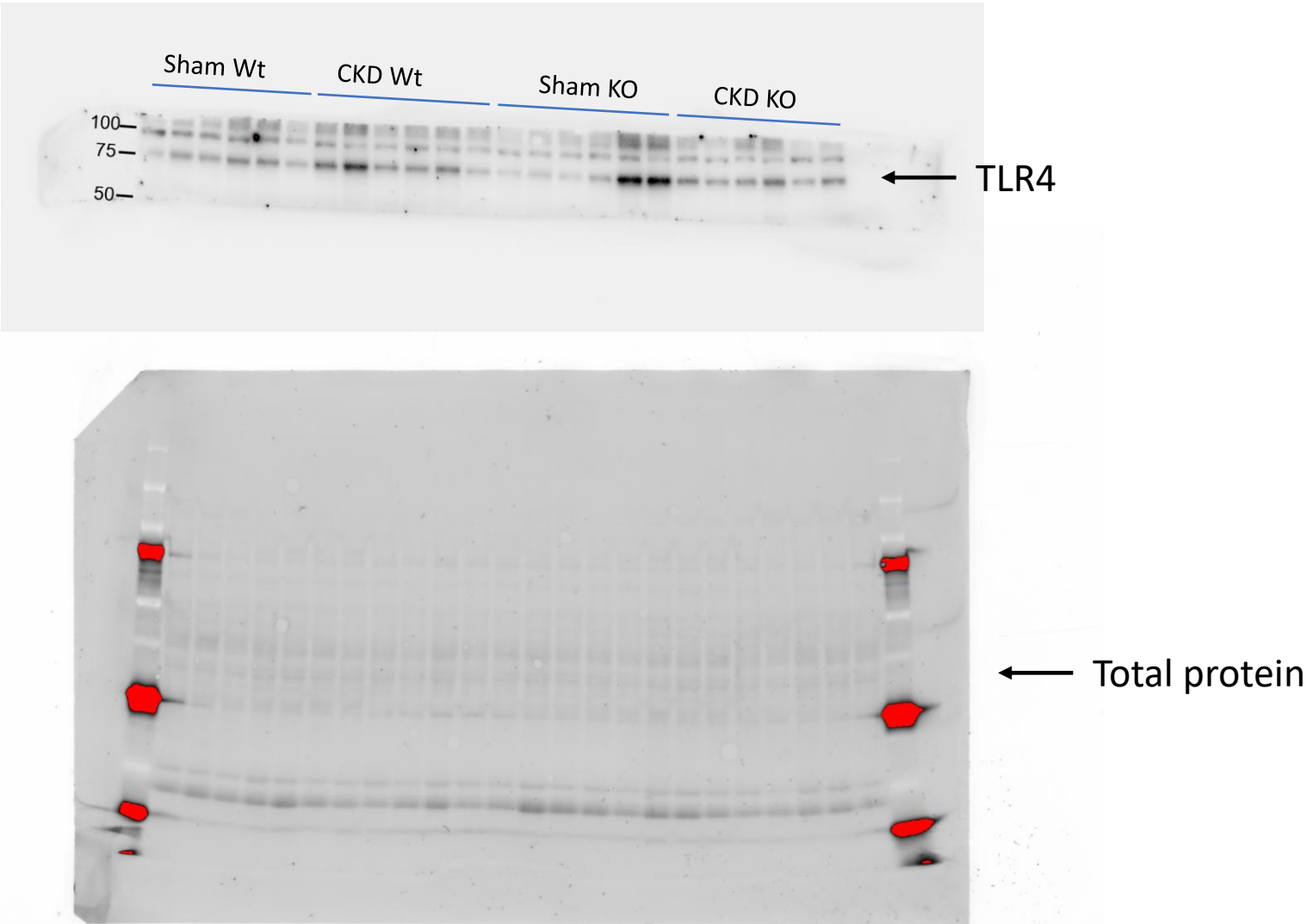

Supplement: Supplementary file 2 [file hyp-83-e25658-s002.pdf]
